# Supplementary material for: Fermented foods affect the seasonal stability of gut bacteria in an Indian rural population
Source: Nat Commun. 2025 Jan 17;16:771. doi: 10.1038/s41467-025-56014-6 (PMC11748640; doi:10.1038/s41467-025-56014-6)
Supplement: Supplementary file 1 — Supplementary Information [file 41467_2025_56014_MOESM1_ESM.pdf]

SUPPLEMENTARY INFORMATION

Title: Fermented foods affect the seasonal stability of gut bacteria in an Indian rural population

Authors: Kumaraswamy Jeyaram, Leo Lahti, Sebastian Tims, Hans G.H.J. Heilig, Antonie H. van Gelder, Willem M. de Vos, Hauke Smidt & Erwin G. Zoetendal

1. Supplementary Methodology and Results:

1.1 Details of subject enrollment and summary of the results obtained from the questionnaire administered to the Indian study population about their dietary and lifestyle habits during the seasonal sampling

Subject enrollment details:

We surveyed with a questionnaire on the consumption frequency and quantity of two selected fermented foods (*Hawaijar* and *Dahi*) and collected information from 1104 people in Imphal Valley, Manipur, India. Initial screening was done with the eligibility criteria of good general health without any chronic illness, not taking antibiotics within six months, normal bowel frequency, and free from any gastrointestinal disorders. The persons who consume more than three times/ week to daily the above-fermented foods, and have not consumed the above-fermented foods at least for the last ten years were categorised. With the above criteria, ~20 subjects each in four diet groups were targeted, and a total of 85 subjects were enrolled for the study. Among the 85 targeted subjects, seven were dropped out (three declined during the sample collection and, four experienced health issues after the first sampling due to their health condition, medication/antibiotic intake or deviation from the eligibility criteria. Seven seasonal samples were missed due to the subject's travel during the collection time. The samples from 78 out of 85 enrolled subjects were considered for the study.

Summary of the results obtained from the questionnaire administered to the Indian study population about their dietary and lifestyle habits during the seasonal sampling

Though the Meitei community of Manipur state (India) is traditionally a fish-eating vegetarian, the study population was mostly non-vegetarian (~87%), and only 13% of the subjects were fish-eating vegetarians. They consume two meals per day, with rice as a staple food. Fish, chicken, and duck were the common meat items they consumed. The study population mostly drink the government-processed tap water supply in the region. All consume tea as a regular beverage, and only 16% prefer coffee. Additional lifestyle habits of chewing areca nuts with betel leaf and occasional alcohol drinking were recorded in nearly 41% and 20%, respectively, in the subjects. The study population belonged to the Mangang, Luwang, Khuman, Khaba-Nganba and Moirang clans of the Meitei community. These clans are strictly exogamous, whereas the overall Meitei community is endogamous with a similar food culture. No major illness was reported during the sampling, and none had taken antibiotics at least six months before sampling. The selected subjects were balanced with sex ratio, age (15-64 years) and BMI (18.5-30.0) in the categorised groups (Suppl. Table S1). All the subjects (except 2) were born vaginally and received breastfeeding in childhood. The reported staple foods were rice, pulses, seasonal vegetables and mushrooms. Among the seasonal vegetables, mustard leaves, cabbage and cauliflower were consumed mostly in the winter. Two days' diet recall showed the consumption pattern of mustard leaves (2%, 27%, 4 5%), cabbage (3%, 6%, 37%) and cauliflower (0%, 1%, 16%) during different seasons (summer, autumn, winter) respectively. Among the unique vegetables consumed here, *Hibiscus sabdariffa* leaf was consumed mostly in summer (~20%) and *Parkia speciosa* pods during winter (~6%).

1.2 Methodology for assessing the bacterial community structure of fermented foods (*Hawaijar* and *Dahi*) by Illumina-MiSeq amplicon sequencing

Bacterial community structure of fermented foods (*Hawaijar* and *Dahi*)

DNA extraction:

DNA of *Dahi* (fermented milk) and *Hawaijar* (fermented soybean samples) were extracted by the method described by Keisam et al. (2016)[1], by combining bead beating (zirconia/silica beads) and enzymatic lysis (lysozyme, mutanolysin and proteinase-K), purification with chloroform: isoamyl alcohol and precipitation with isopropanol. The purity and quantity of the DNA extracted were assessed using a spectrophotometer (NanoDrop ND-1000, USA), and stored at −20 °C for further analysis.

Barcoded Illumina MiSeq Sequencing and data processing:

The primers with barcodes targeting the V4-V5 region of the 16S rRNA gene, F563–577 (5′-AYTGGGYDTAAAGNG-3′) and R924–907 (5′-CCGTCAATTCMTTTRAGT-3′) were used for the amplicon sequencing as described earlier[2]. The PCR products were purified using the QIAquick gel extraction kit (Qiagen, New Delhi, India) and quantified using the Qubit Assay Kit (Invitrogen). The multiplexed amplicon pool was sequenced in the Illumina MiSeq platform (Xcelris, Ahmedabad), and the raw sequence data were processed through the QIIME 2 bioinformatics pipeline[3,] and the SILVA release 138 database was used for the generation of OTU tables at different taxonomic levels. The sequence data were available in the NCBI-SRA (accession number: PRJNA1191989).

Supplementary Figures, Figure S1

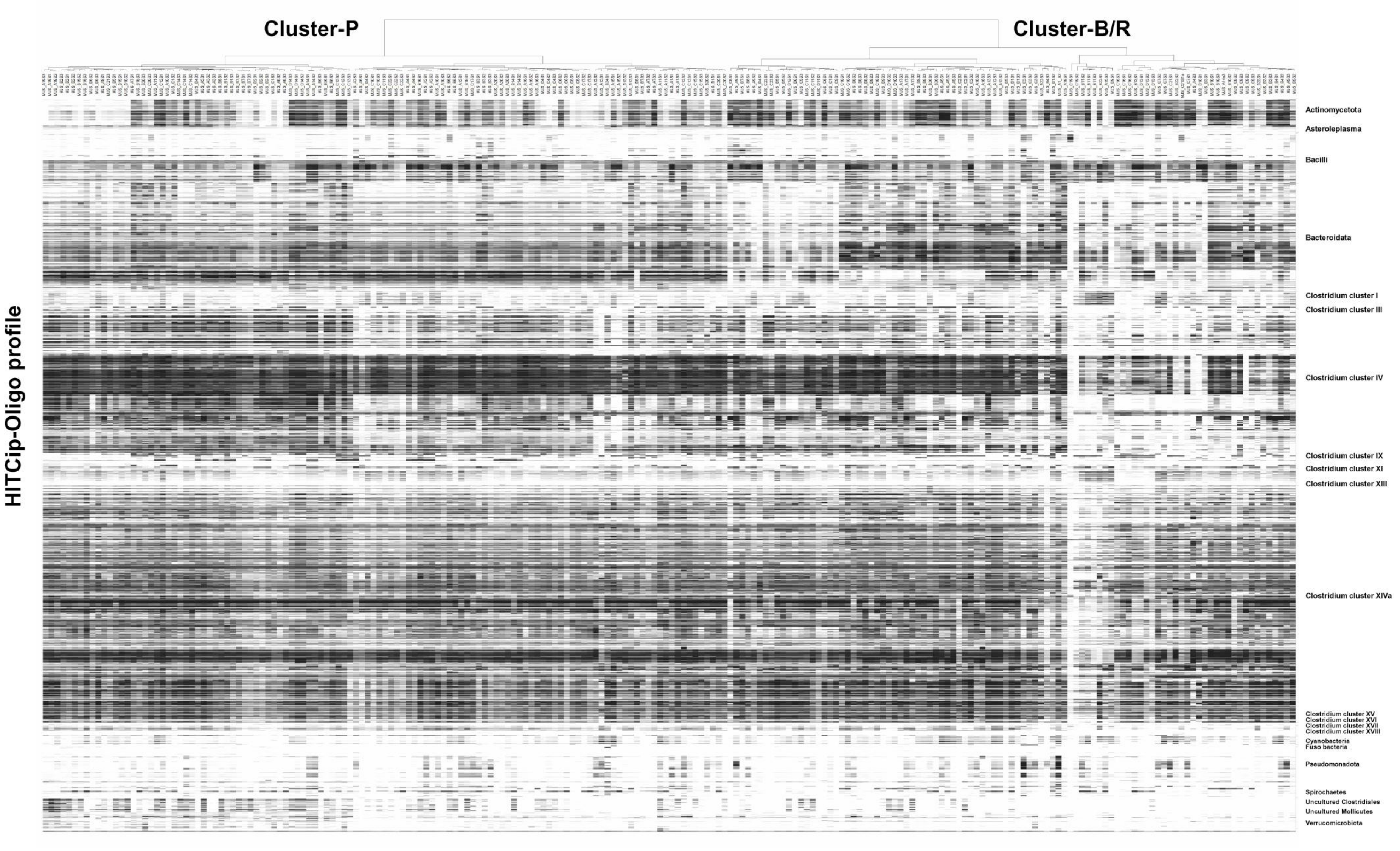

Figure S1. HITChip oligo profile-based grouping of gut microbiota composition of the Indian study population resulted in two distinct clusters (complete hierarchical clustering of the log10 transformed HITChip probe signal oligo profile with Pearson similarity). The two clusters formed from the HITChip probe profile (n=214) are named here as *Prevotella*-driven Cluster-P and *Bifidobacterium*/*Ruminococcus*-driven Cluster-B/R. The intensity of the heat map relates to the relative abundance of the major bacterial phylum, as shown in the legend.

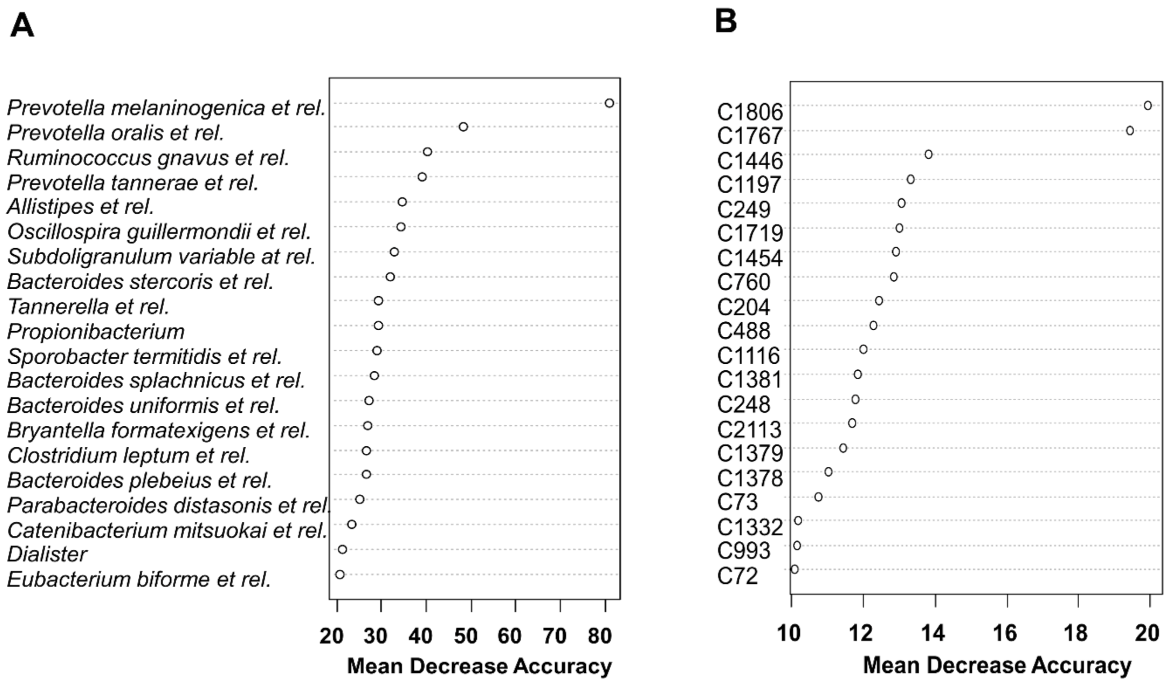

Figure S2 Random Forest analysis showed the top 20 key signature taxa (A) and metabolites (B) that best differentiate between the two clusters of gut microbiota (*Prevotella*-driven cluster-P, n=117 and *Bifidobacterium/Ruminococcus*-driven cluster-B/R, n=97) observed in the Indian study population. *Prevotella melaninogenica* et rel. as the key gut bacterial driver for separating these two clusters (~ 80 Mean decrease accuracy); and the long-chain fatty acid derivatives C1767 (a derivative of pentadecanoate) and C1806 (2-hexadecanoyl-sn-glycero-3-phosphoethanolamine) as the key differentiating metabolites (~ 20 Mean decrease accuracy) between the two clusters (see the Supplementary Table S3). Source data are provided as a Source Data file.

Figure S3

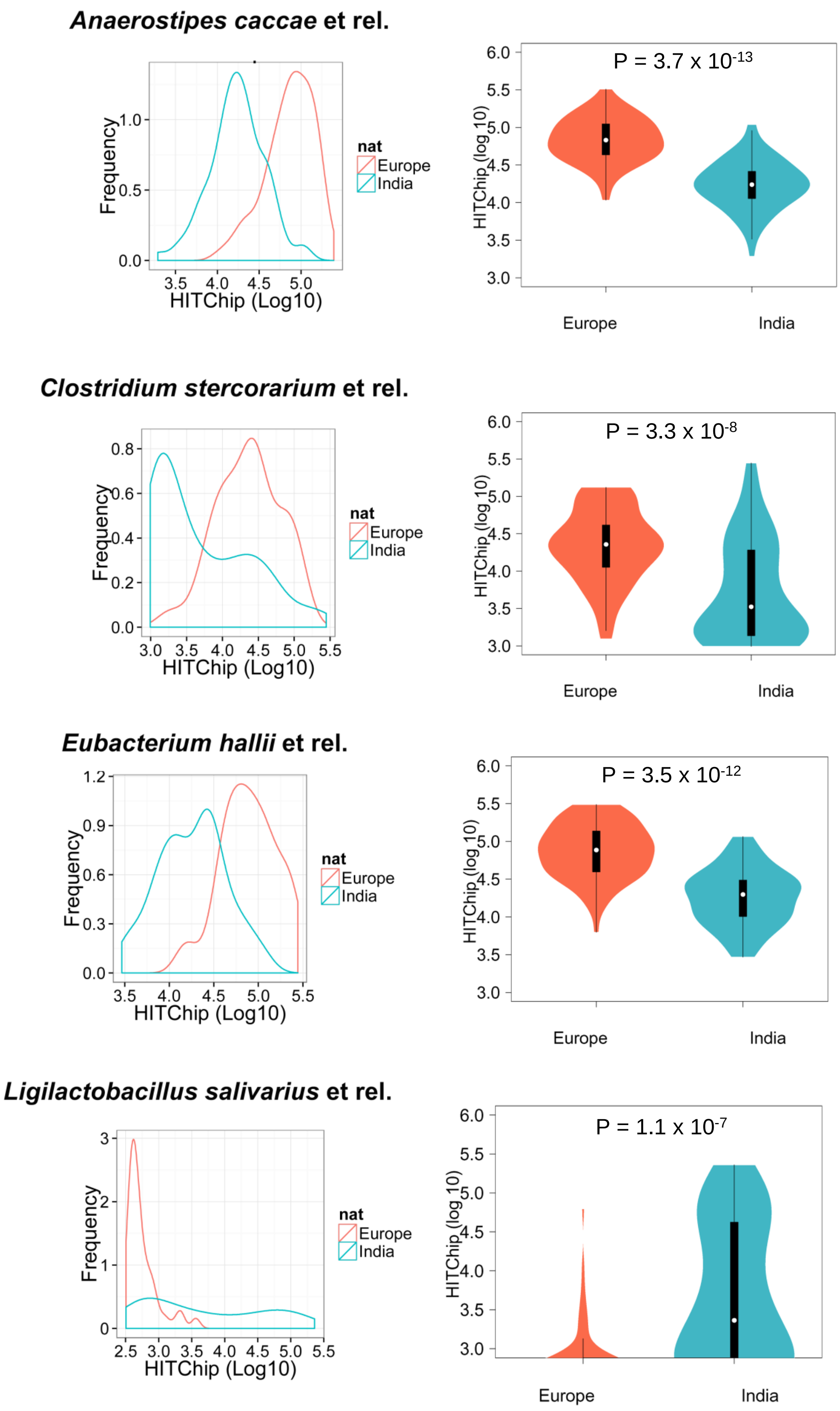
Anaerostipes caccae et rel.
Frequency
HITChip (Log10)
nat
Europe
India
P = 3.7 x 10-13
HITChip (log 10)
Europe
India
Clostridium stercorarium et rel.
Frequency
HITChip (Log10)
nat
Europe
India
P = 3.3 x 10-8
HITChip (log 10)
Europe
India
Eubacterium hallii et rel.
Frequency
HITChip (Log10)
nat
Europe
India
P = 3.5 x 10-12
HITChip (log 10)
Europe
India
Ligilactobacillus salivarius et rel.
Frequency
HITChip (Log10)
nat
Europe
India
P = 1.1 x 10-7
HITChip (log 10)
Europe
India

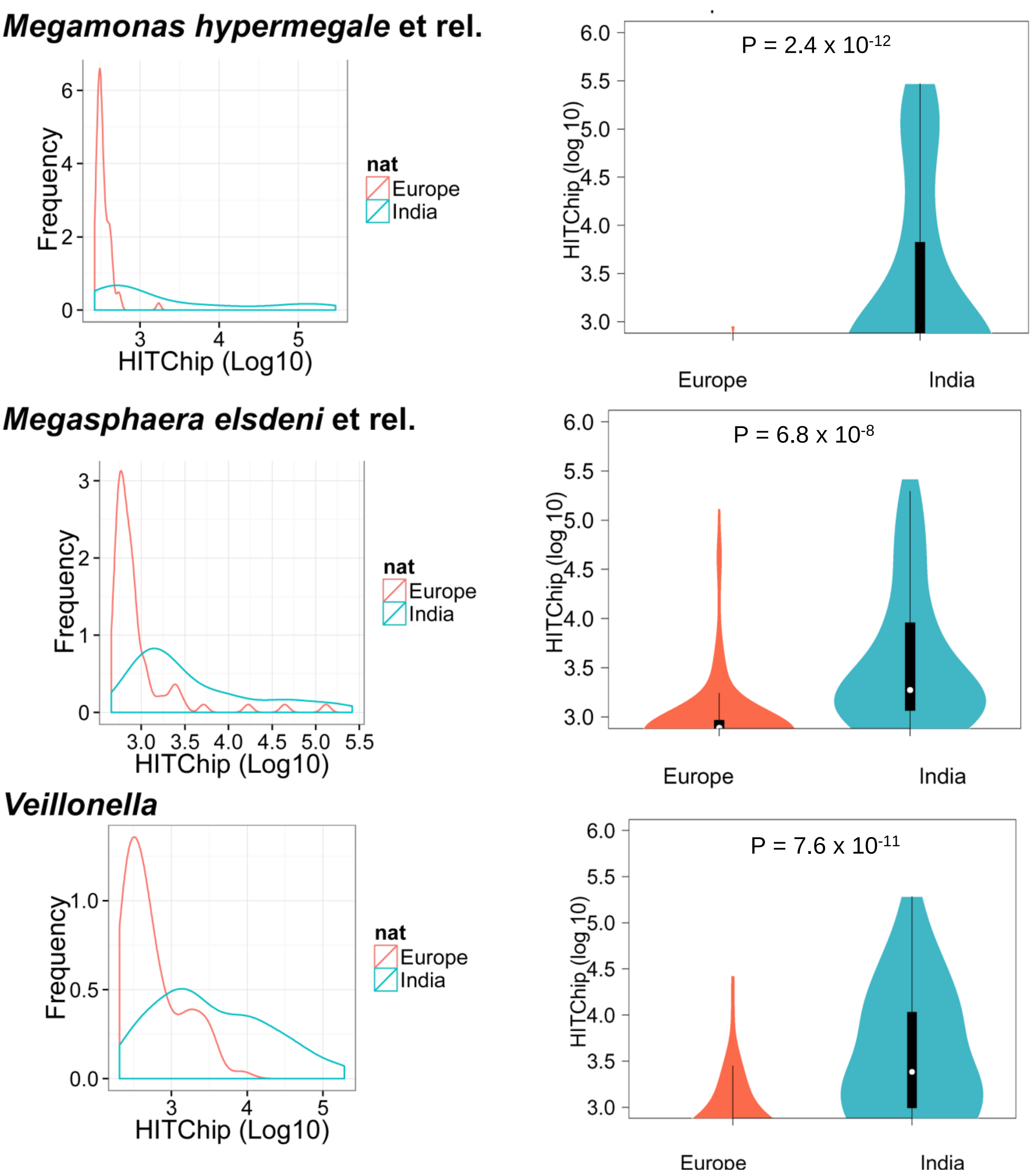

Figure S3 The abundance distribution for the taxa that are significantly high in European (n=76) and Indian subjects (n=76) are visualised here ($p < 0.05$, two-sided Wilcoxon test, Benjamini-Hochberg method adjusted; > 2 absolute fold-change of HITChip signal). The density plots and the matched line plots show the comparison between the Indian and European samples. The violin and whisker plots display the median (middle point), and the box ranges from 25 to 75 percentile with a whisker of lower than 1.5 IQR. The statistical significance of the difference between the biological replicates of Indian (n=76) and European (n=76) samples

was analysed by a two-sided paired Wilcoxon test, and the p-values are indicated in the figure panel. The colour key for Europeans is orange-red and Indian in blue-green in all the figure panels. Source data are provided as a Source Data file.

.

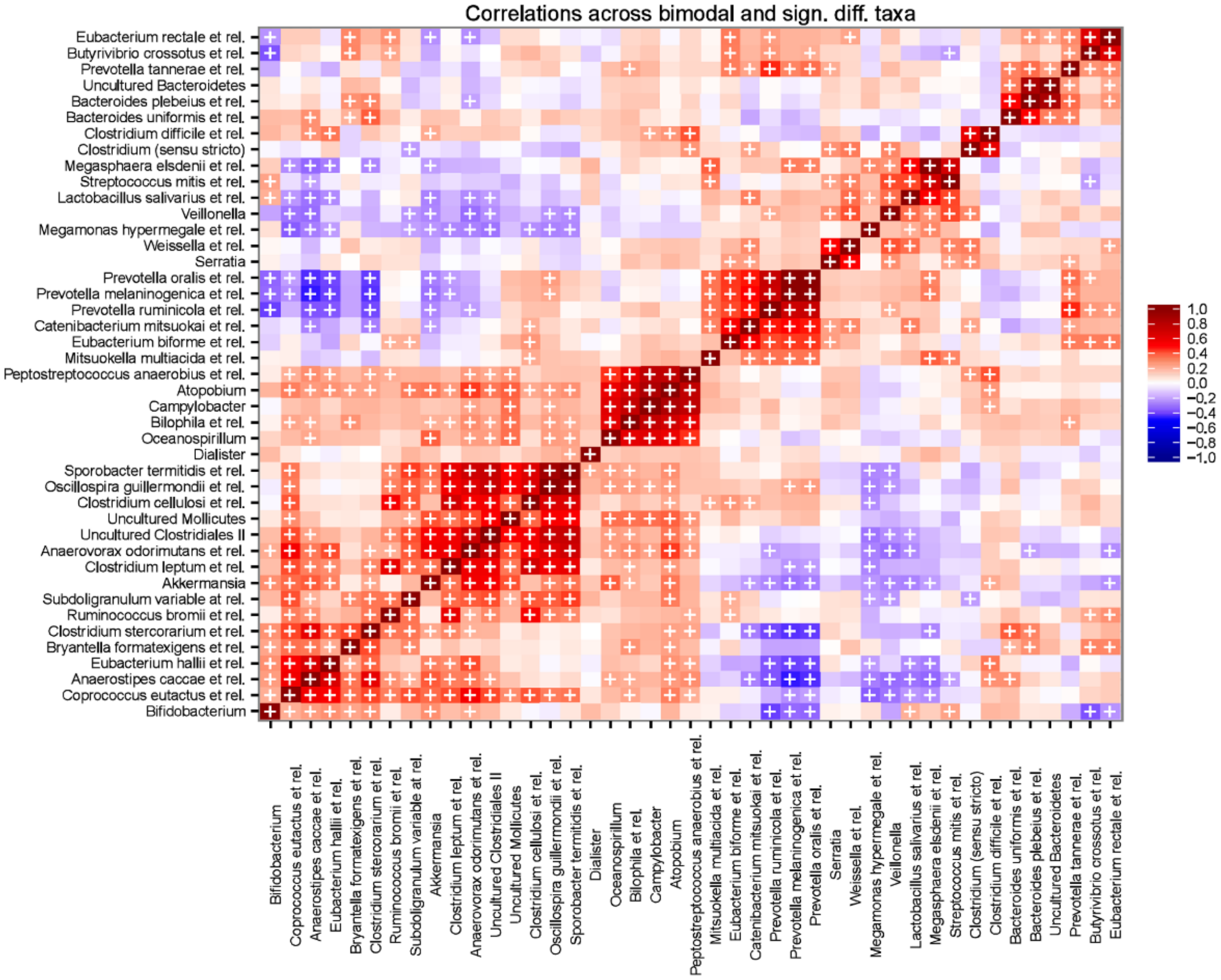

Figure S4 The correlation heat map shows the Spearman correlation coefficient across all samples (European, n=76 and Indian, n=76) between the taxa that are either significantly different between European and Indian (bimodal or unimodal) or bimodal within Indians or Europeans (but not necessarily significantly different). The significance in the correlation coefficient was calculated by the two-sided Wilcoxon test with Bonferroni correction and marked with + in the heat map.

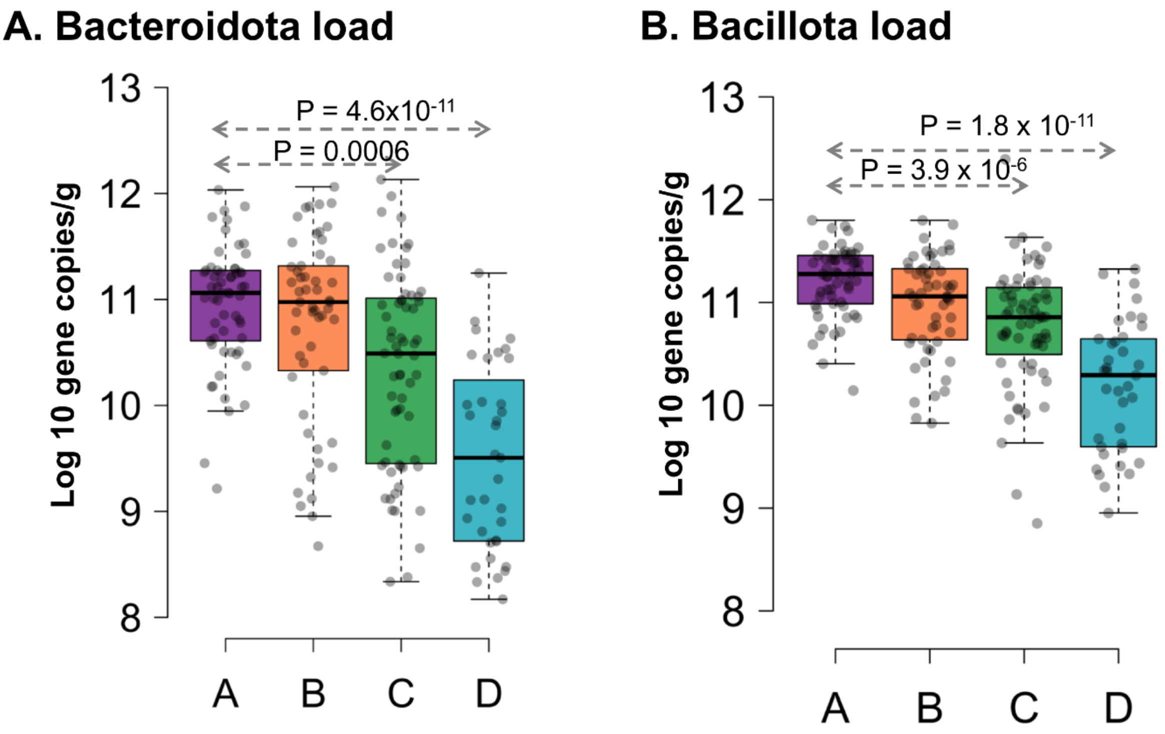

Figure S5. Effect on the Bacteroidota and Bacillota load during long-term fermented foods consumption. The box plots show a lower Bacteroidota and Bacillota load in the fermented food-consuming groups (C and D), quantified by phylum-specific qPCR assay and expressed as 16S rRNA gene copies/g of wet faeces. The diet groups are Group-A (n=58): not consumed *Dahi* and *Hawaijar*, Group-B (n=54): consumed *Dahi* and *Hawaijar*, Group-C (n=65): consumed *Hawaijar*, not *Dahi*, and Group-D (n=37): consumed *Dahi*, not *Hawaijar*, The box and whisker plots display the median (middle line), and the box ranges from 25 to 75 percentile with Tukey whisker (less than 1.5 IQR). The statistical significance of variation between the study groups was calculated by ANOVA and Bonferroni corrected and indicated as p-value in the figure panels. Source data are provided as a Source Data file.

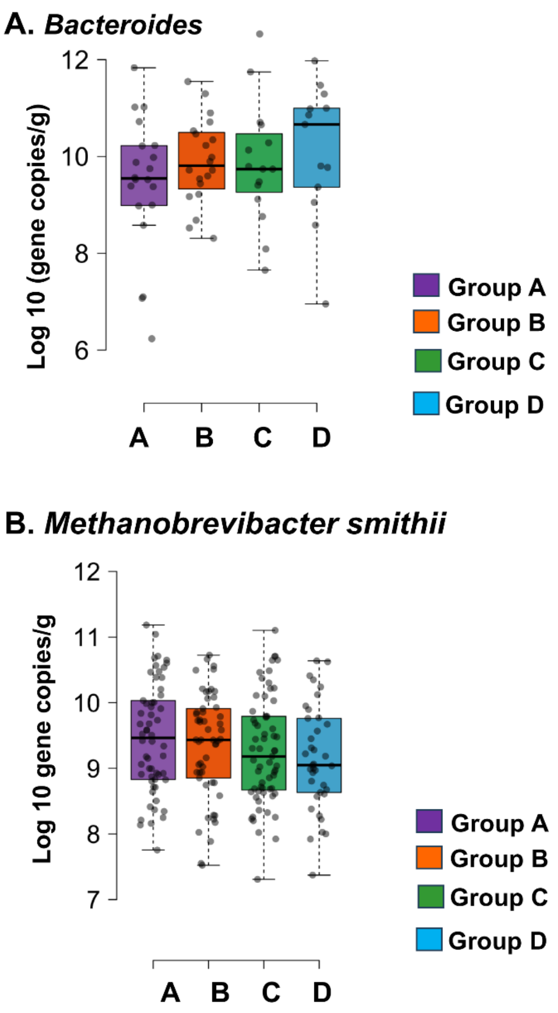

Figure S6 Box plots show no relevant difference in the absolute load of *Bacteroides* (A) and *Methanobrevibacter smithii* (B) observed between the study groups. The absolute bacterial load was analysed by group-specific qPCR assay and expressed as 16Sr RNA gene copies/g of wet faeces in between the categorised groups. The sample numbers for *Methanobrevibacter smithii* load in each diet group are Group-A: n=58, Group-B: n=54: Group-C: n=65 and Group-D: n=37. The sample numbers for *Bacteriodes* load in each diet group are Group-A: n=20, Group-B: n=20: Group-C: n=15 and Group-D: n=13. The box and whisker plots display the median (middle line), and the box ranges from 25 to 75 percentile with Tukey whisker (less than 1.5 IQR). No statistical significance of variation between the study groups was observed between the study groups (two-sided Wilcoxon test).). Source data are provided as a Source Data file.

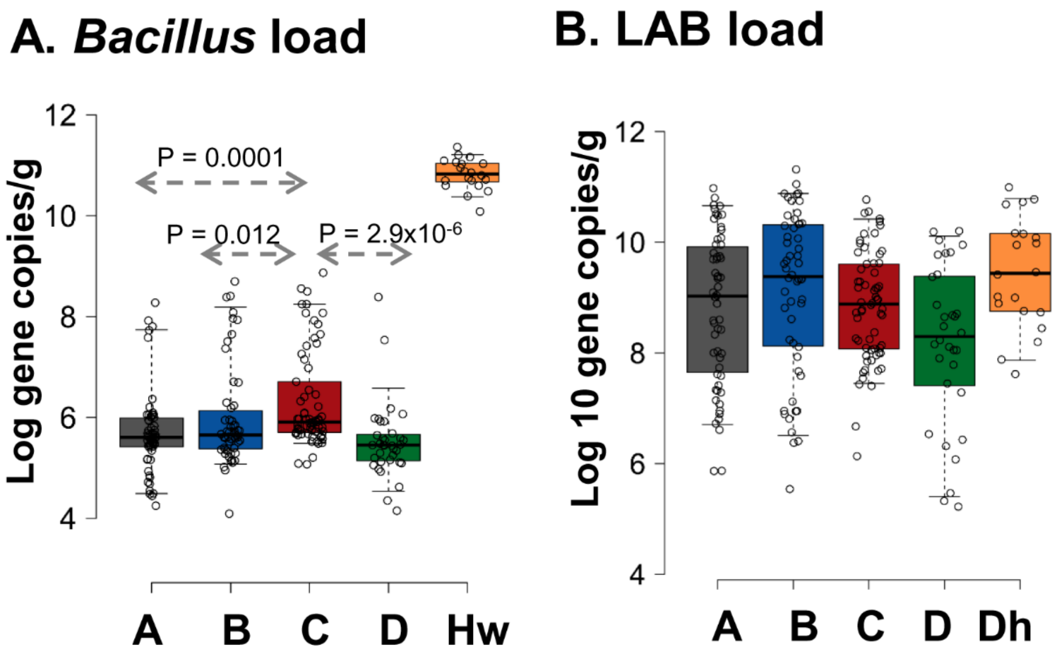

Figure S7 Effect on the ingested bacterial load during long-term consumption of fermented foods. Box plots show the difference in *Bacillus* (**A**) and lactic acid bacterial load (**B**) in the faecal samples of the study groups and the fermented foods**.** The diet groups are Group-A (n=58): not consumed *Dahi* and *Hawaijar*, Group-B (n=54): consumed *Dahi* and *Hawaijar*, Group-C (n=65): consumed *Hawaijar*, not *Dahi*, and Group-D (n=37): consumed *Dahi*, not *Hawaijar*. *Bacillus* and lactic acid bacterial (LAB) loads were analysed by group-specific qPCR and expressed as 16S rRNA gene copies/g of wet faeces**.** 'Hw' indicates the *Bacillus* population/g wet weight in *Hawaijar* samples (n=20), and 'Dh' indicates the LAB population/ml in *Dahi* samples (n=20). The box and whisker plots display the median (middle line), and the box ranges from 25 to 75 percentile with Tukey whisker (less than 1.5 IQR). The significant changes in comparison to the control group were calculated by ANOVA and Bonferroni corrected and indicated as p-value in the figure panels. Source data are provided as a Source Data file.

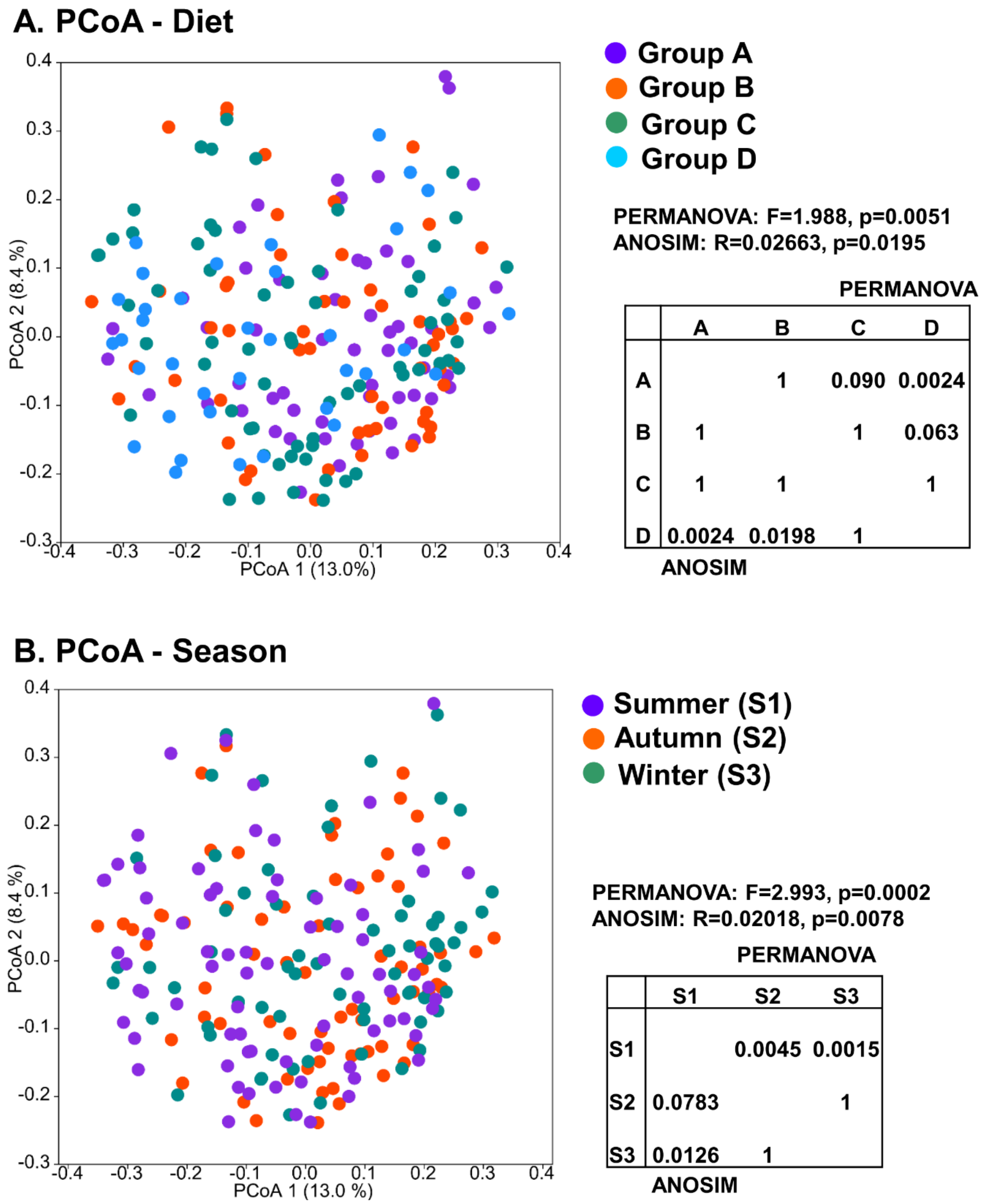

PERMANOVA (upper) / ANOSIM (lower)

	A	B	C	D
A		1	0.090	0.0024
B	1		1	0.063
C	1	1		1
D	0.0024	0.0198	1	

PERMANOVA (upper) / ANOSIM (lower)

	S1	S2	S3
S1		0.0045	0.0015
S2	0.0783		1
S3	0.0126	1	

Figure S8 PCoA scatter plot of Bray-Curtis dissimilarity based on the genus level data using the first two principal components shows the placement of four study groups (A) and three seasons (B) in the Indian study population (n=214). PERMANOVA with 9999 permutations and ANOSIM analysis using Bray-Curtis distances show the overall significance and pairwise significance as Bonferroni corrected p-value indicated in the plot. Source data are provided as a Source Data file.

Figure S9

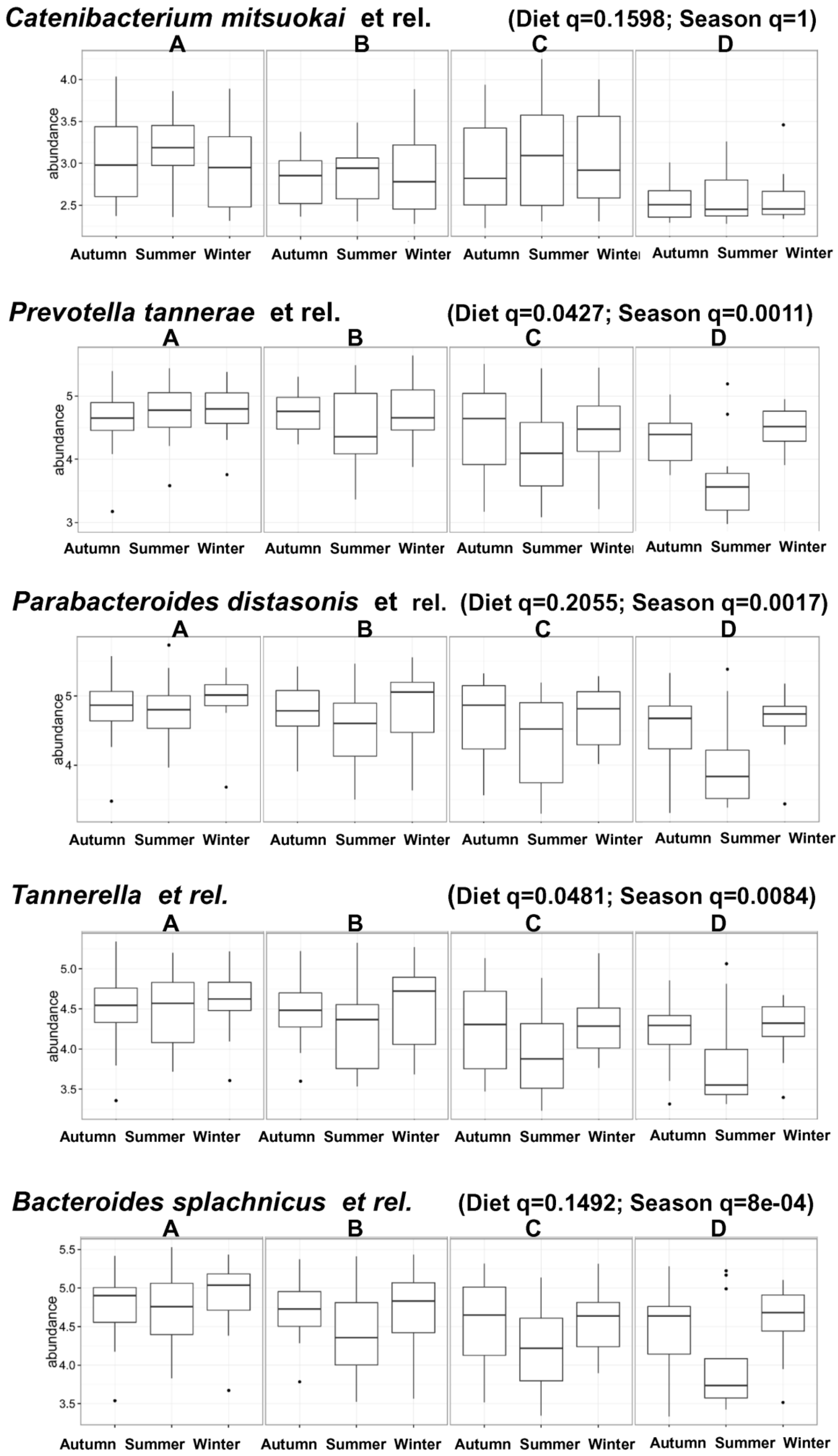
Catenibacterium mitsuokai et rel.
(Diet q=0.1598; Season q=1)
Prevotella tannerae et rel.
(Diet q=0.0427; Season q=0.0011)
Parabacteroides distasonis et rel.
(Diet q=0.2055; Season q=0.0017)
Tannerella et rel.
(Diet q=0.0481; Season q=0.0084)
Bacteroides splachnicus et rel.
(Diet q=0.1492; Season q=8e-04)
A
B
C
D
abundance
Autumn
Summer
Winter

Figure S9 The bacterial taxa with significant diet association after taking into account the season effects (based on the linear mixed-effect model with subject as a random effect, diet and season as fixed effects, Tukey post hoc test of the linear mixed model). Four diet groups are Group-A: not consumed *Dahi* and *Hawaijar* (n=58), Group-B: consumed *Dahi* and *Hawaijar* (n=54), Group-C: consumed *Hawaijar*, not *Dahi* (n=65), and Group-D: consumed *Dahi*, not *Hawaijar*(n=37), and three seasons are S1- Summer (n=76), S2- Autumn (n=70), S3-Winter (n=68). The Box and whisker plots display median (middle line), box ranges from 25 to 75 percentile with Tukey whiskers and outlier (more than 1.5 IQR). The statistical significance of the difference between the diet and season groups with FDR < 0.25, Benjamini-Hochberg method corrected q values are visualised here. Source data are provided as a Source Data file.

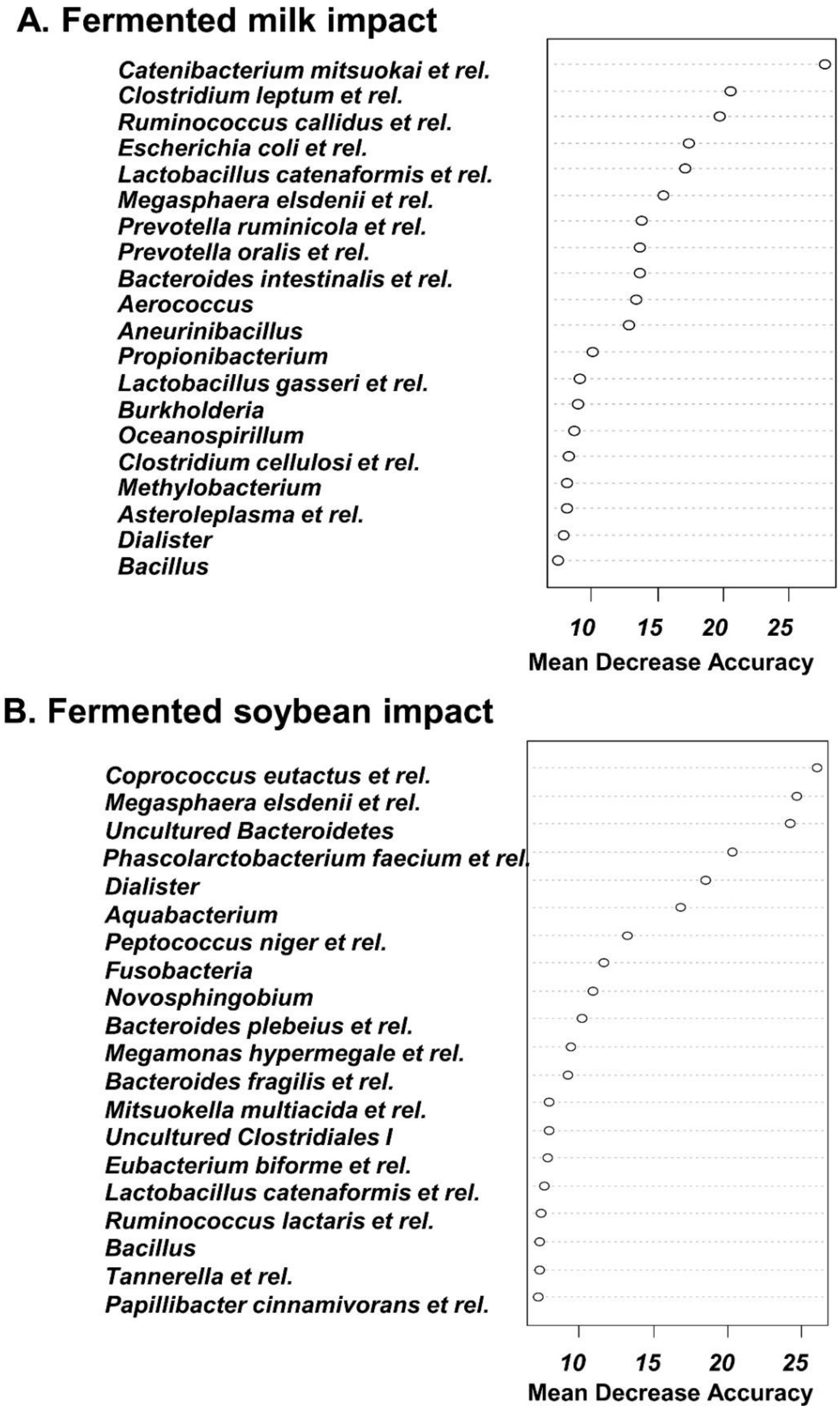

Figure S10. Random Forest analysis showed the top 20 key differentiating taxa due to *Dahi* consumption **(**A) (consumer n=91, control n=123) and *Hawaijar* consumption (B) (consumer n=119, control n=95) in the Indian study population. Source data are provided as a Source Data file.

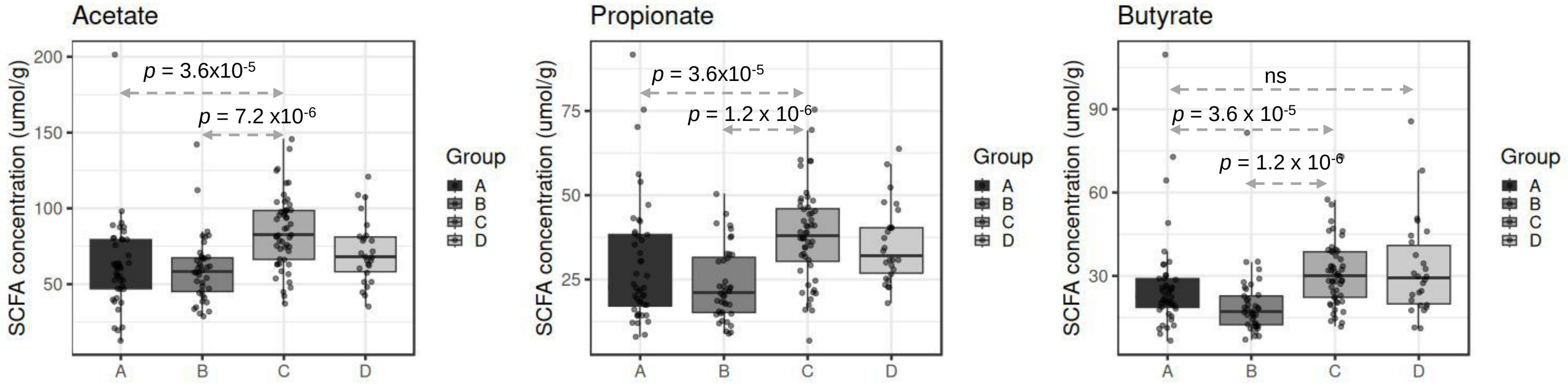

Figure S11 Effect on the short-chain fatty acids (SCFA) composition in different diet groups differ in fermented foods consumption. The box plots show the difference in major SCFA production composition between the study groups (A, B, C & D). The diet groups are Group-A (n=44): not consumed *Dahi* and *Hawaijar*, Group-B (n=40): consumed *Dahi* and *Hawaijar*, Group-C (n=52): consumed *Hawaijar*, not *Dahi*, and Group-D (n=26): consumed *Dahi*, not *Hawaijar*. The Box and whisker plots display median (middle line), box ranges from 25 to 75 percentile with Tukey whiskers and outlier (more than 1.5 IQR). The significant changes between the groups were calculated by a two-sided Wilcoxon test, and the significant p-values are mentioned in the figure panel. Source data are provided as a Source Data file.

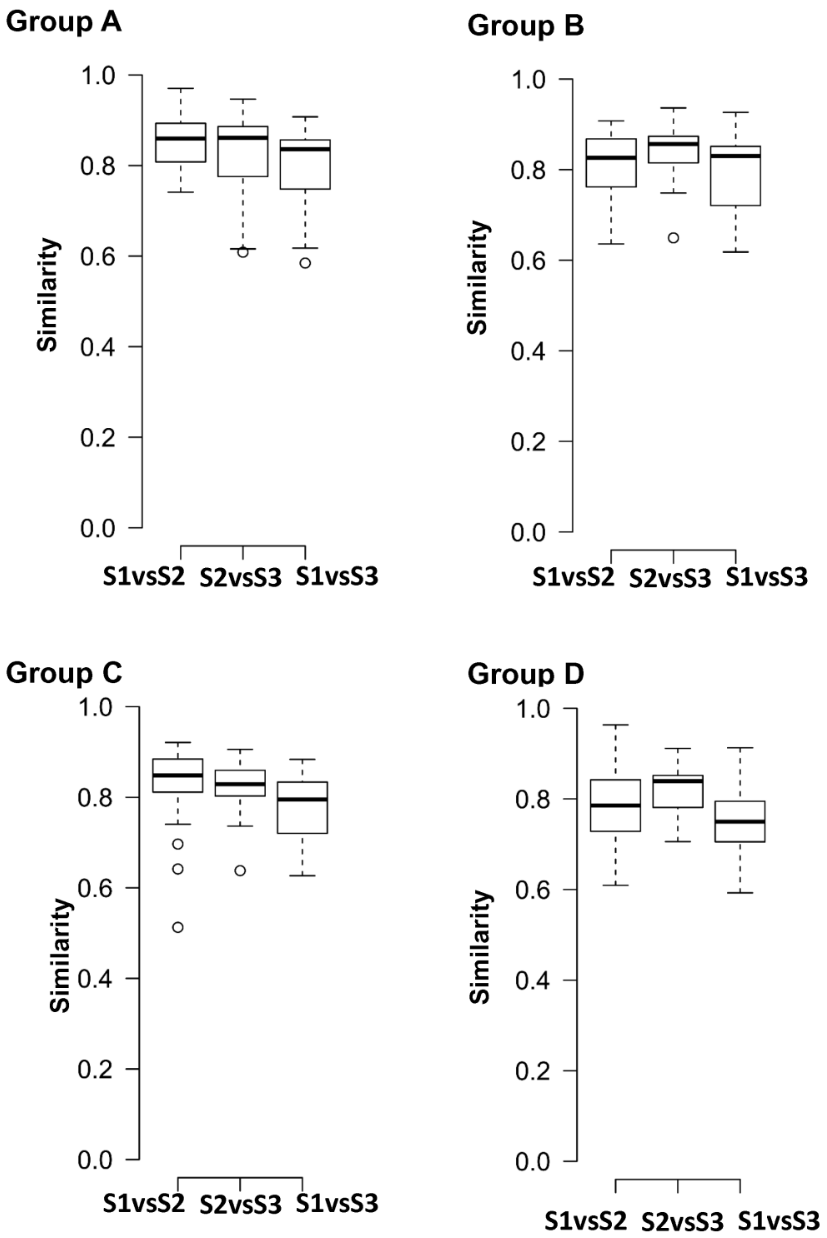

Figure S12 Seasonal intra-individual changes in the gut bacterial similarity in the study groups**.** The diet groups are Group-A: not consumed *Dahi* and *Hawaijar*, Group-B: consumed *Dahi* and *Hawaijar*, Group-C: consumed *Hawaijar*, not *Dahi*, and Group-D: consumed *Dahi*, not *Hawaijar*. The similarity was calculated by comparing the HITChip oligo profiles of paired time interval samples (Summer S1, Autumn S2 and Winter S3) using the Pearson correlation coefficient (Group-A: S1vsS2: n=19, S2vsS3: n=19, S1vsS3: n=19; Group-B: S1vsS2: n=18, S2vsS3: n=15, S1vsS3: n=15; Group-C: S1vsS2: n=22, S2 vs S3: n=19, S1vsS3: n=20; Group-

D: S1vsS2: n=12, S2vsS3: n=11, S1vsS3: n=12). The box and whisker plots display median (middle line), box ranges from 25 to 75 percentile with Tukey whisker lower than 1.5 IQR. The box plots show no statistical significance in the gut bacterial similarity change between the time interval samples (S1vsS2, S2vsS3, S1vsS3) in each study group between the seasons (ANOVA). Source data are provided as a Source Data file.

Figure S13

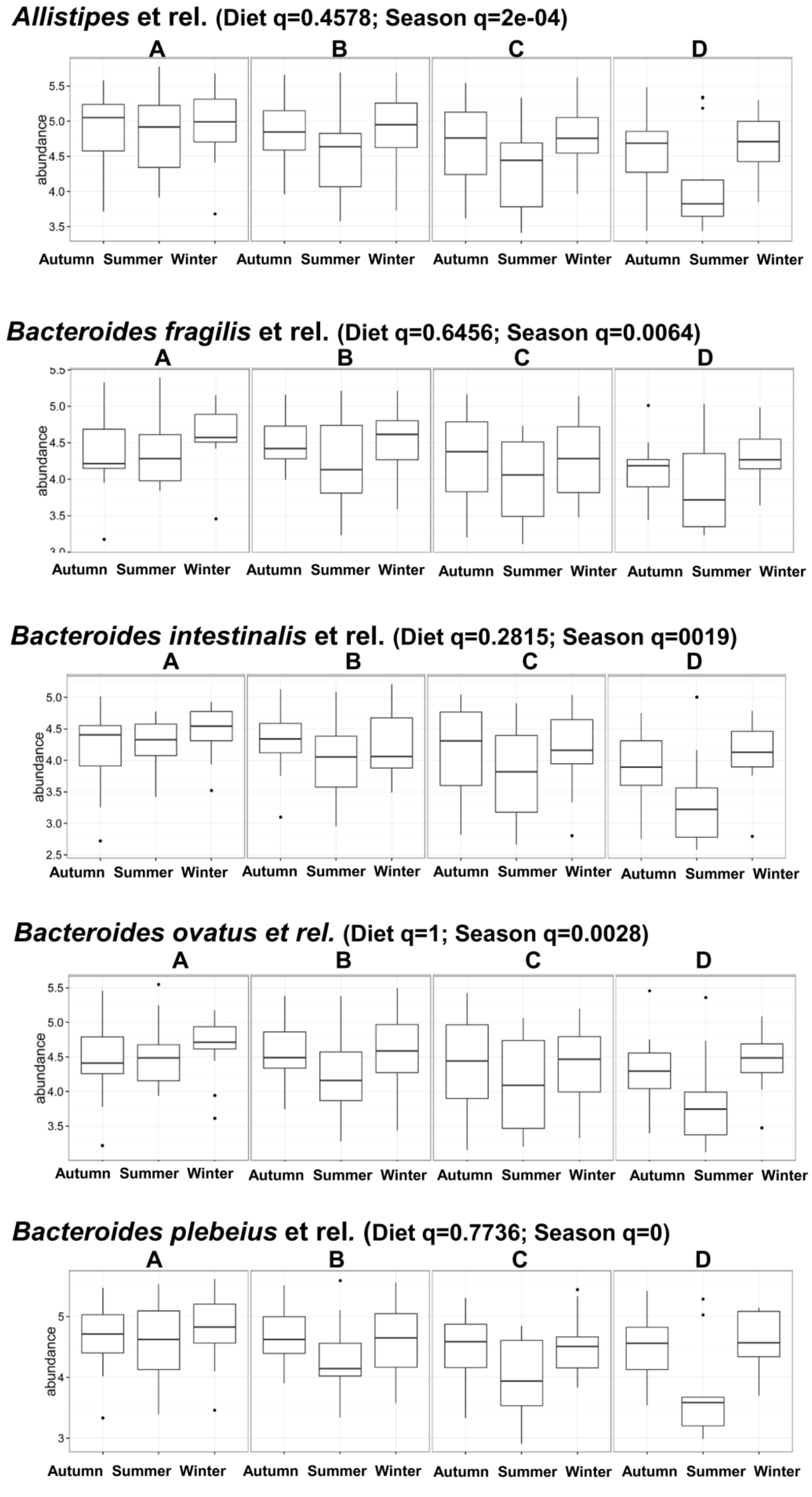
Allistipes et rel. (Diet q=0.4578; Season q=2e-04)
A
B
C
D
abundance
Autumn Summer Winter
Bacteroides fragilis et rel. (Diet q=0.6456; Season q=0.0064)
Bacteroides intestinalis et rel. (Diet q=0.2815; Season q=0019)
Bacteroides ovatus et rel. (Diet q=1; Season q=0.0028)
Bacteroides plebeius et rel. (Diet q=0.7736; Season q=0)

Figure S13 (contd..)

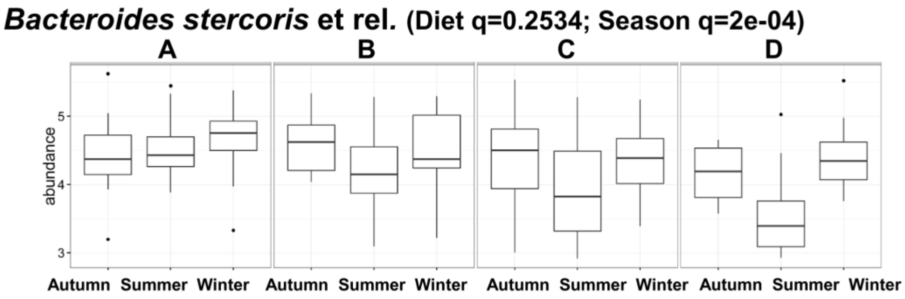

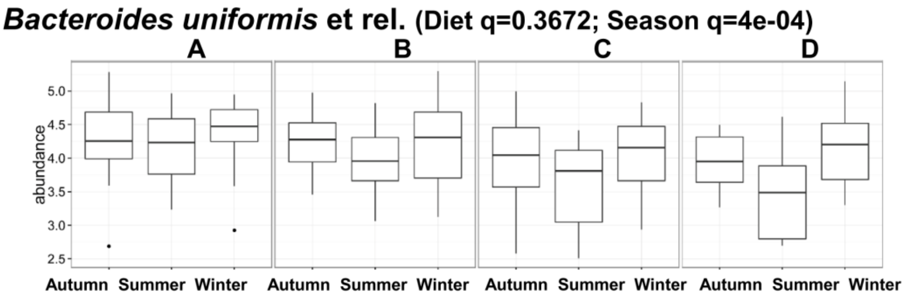

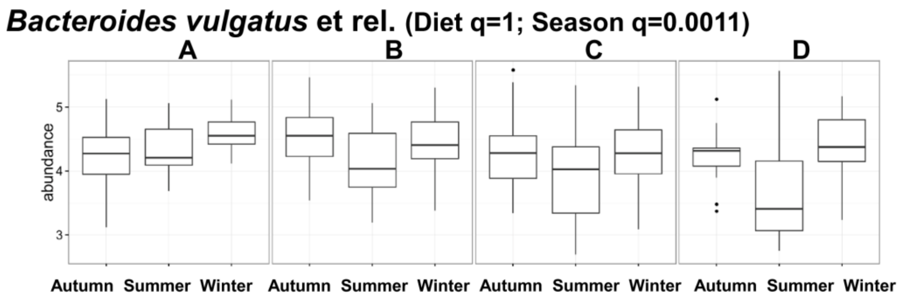

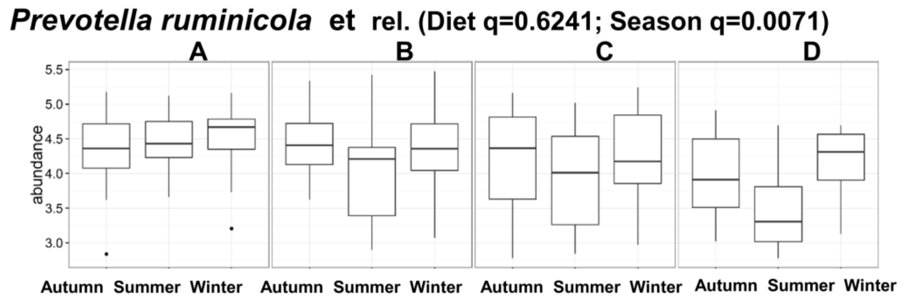

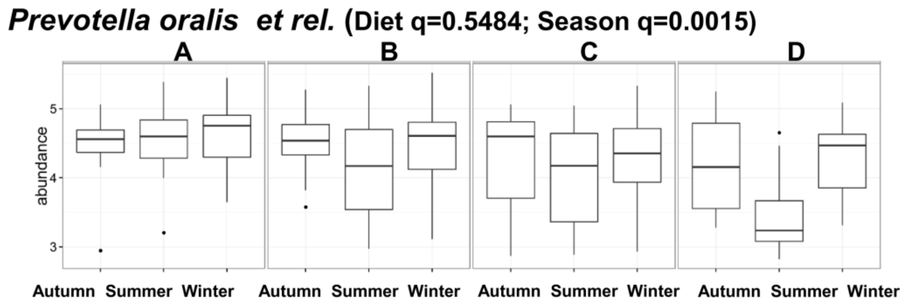

Figure S13 (contd..)

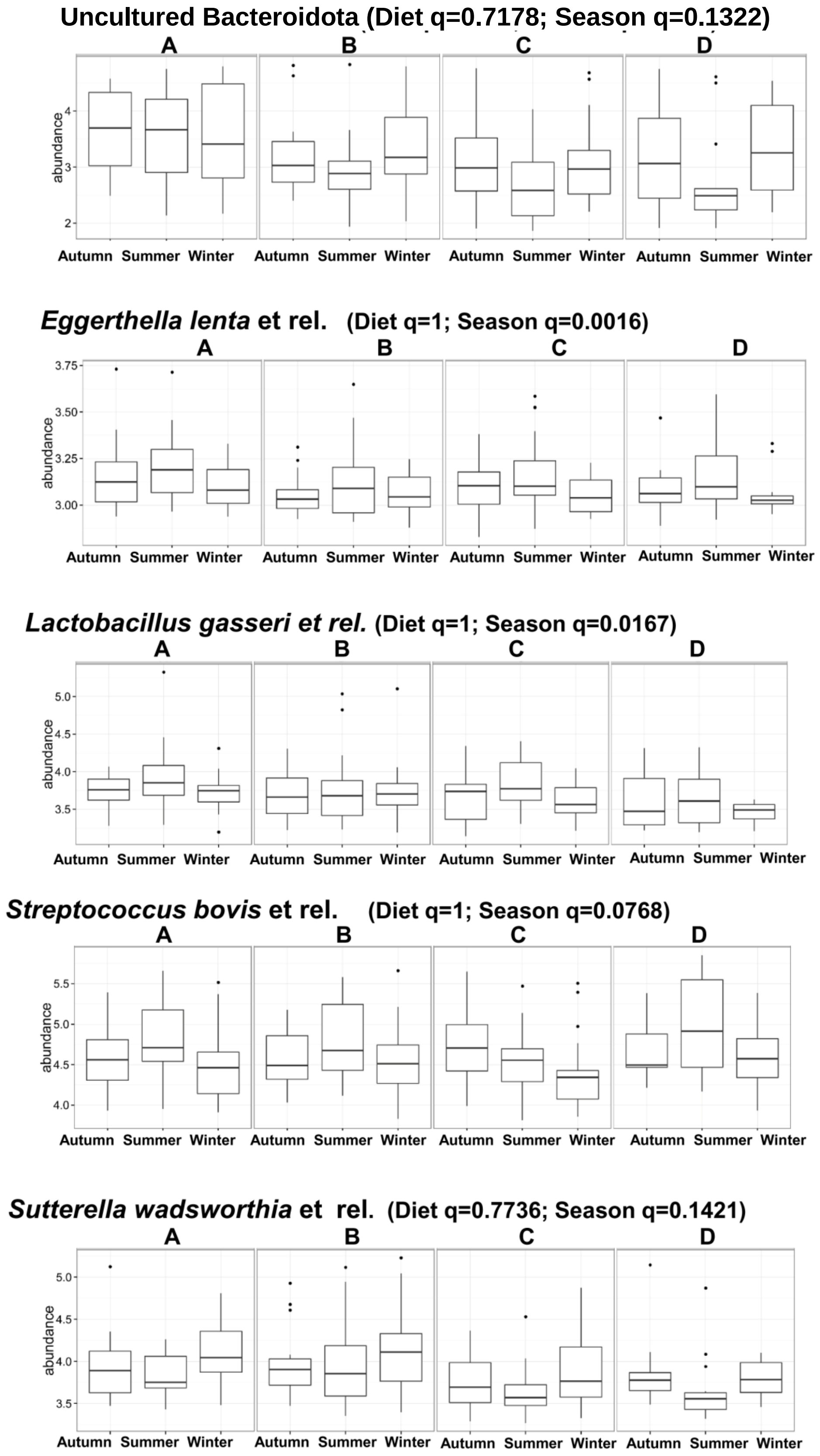

Figure S13. The bacterial taxa with a significant seasonal association after taking into account the diet effect (based on the linear mixed-effect model with subject as a random effect, diet and season as fixed effects, Tukey post hoc test of the linear mixed model). Four diet groups are Group-A: not consumed *Dahi* and *Hawaijar* (n=58), Group-B: consumed *Dahi* and *Hawaijar* (n=54), Group-C: consumed *Hawaijar*, not *Dahi* (n=65), and Group-D: consumed *Dahi*, not *Hawaijar* (n=37), and three seasons are S1- Summer (n=76), S2- Autumn (n=70), S3-Winter (n=68). The Box and whisker plots display median (middle line), box ranges from 25 to 75 percentile with Tukey whiskers and outlier (more than 1.5 IQR). The statistical significance of the difference between the diet and season groups with FDR < 0.25, Benjamini-Hochberg method corrected q values are visualized here. Source data are provided as a Source Data file.

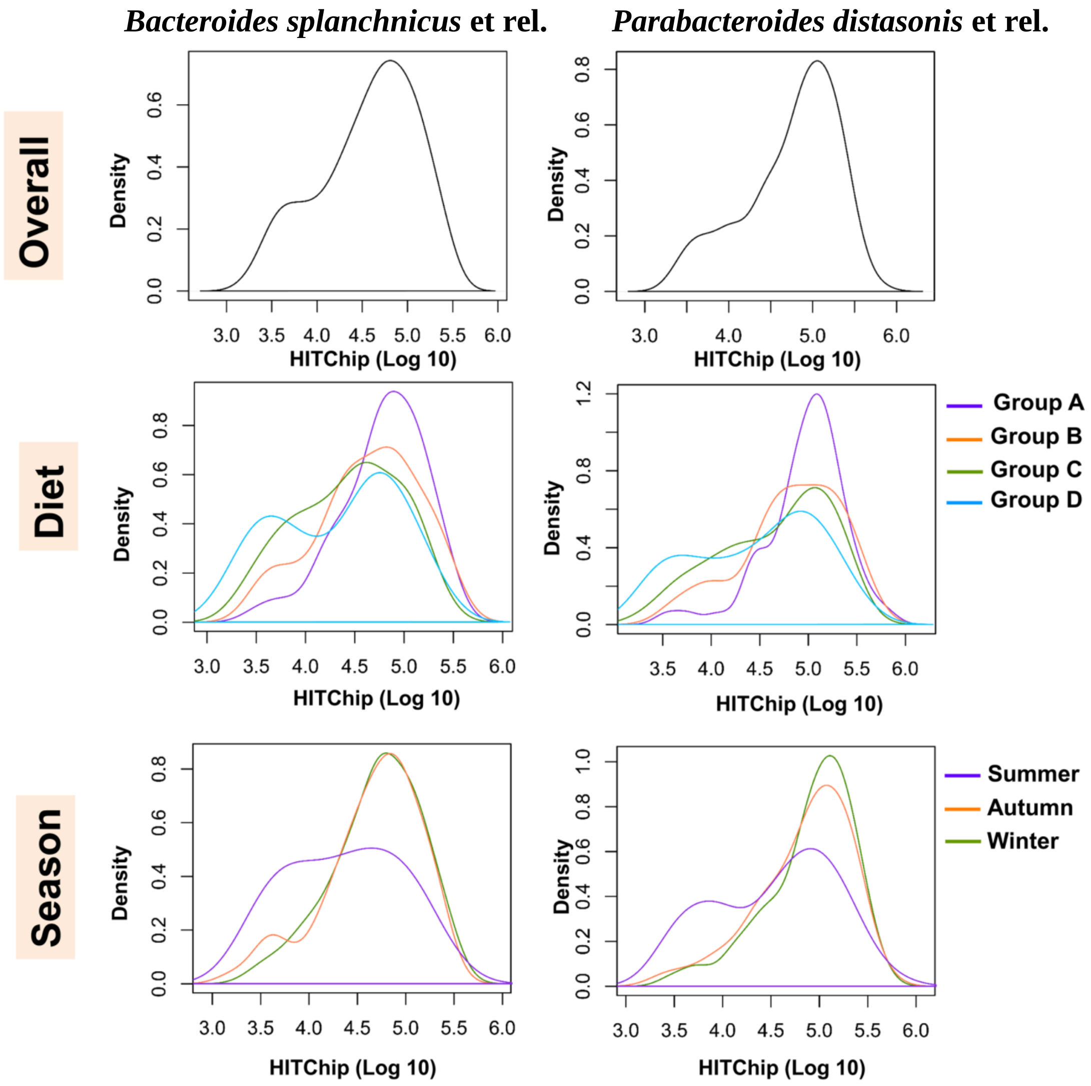

Figure S14. Changes in the bimodal distribution of *Bacteroides splanchnicus* et rel. and *Parabacteroides distasonis* et rel. with significant diet association between four study groups (Diet) and three seasons (Season). The density plots show a shift in the bimodal distribution due to diet and seasonal effects. The diet groups are Group-A: not consumed *Dahi* and *Hawaijar* (n=58), Group-B: consumed *Dahi* and *Hawaijar* (n=54), Group-C: consumed *Hawaijar*, not *Dahi* (n=65), and Group-D: consumed *Dahi*, not *Hawaijar* (n=37), and three seasons are Summer (n=76), Autumn (n=70), and Winter (n=68). The statistical significance of the taxa with diet associations was calculated by Tukey post hoc test of the linear mixed-effect model with false discovery rate FDR<0.25, Benjamini-Hochberg method adjusted p-value. Source data are provided as a Source Data file.

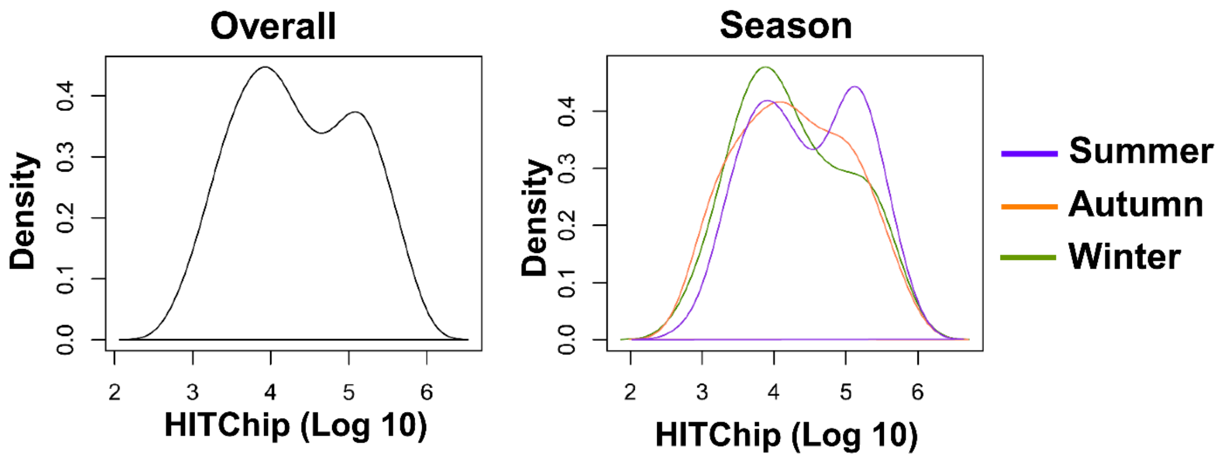

Figure S15 The density plots from HITChip data show a trend of shifting the bimodal distribution of *Bifidobacterium* due to the seasonal effects. The three seasons are Summer (n=76), Autumn (n=70), and Winter (n=68). Source data are provided as a Source Data file.

Figure S16

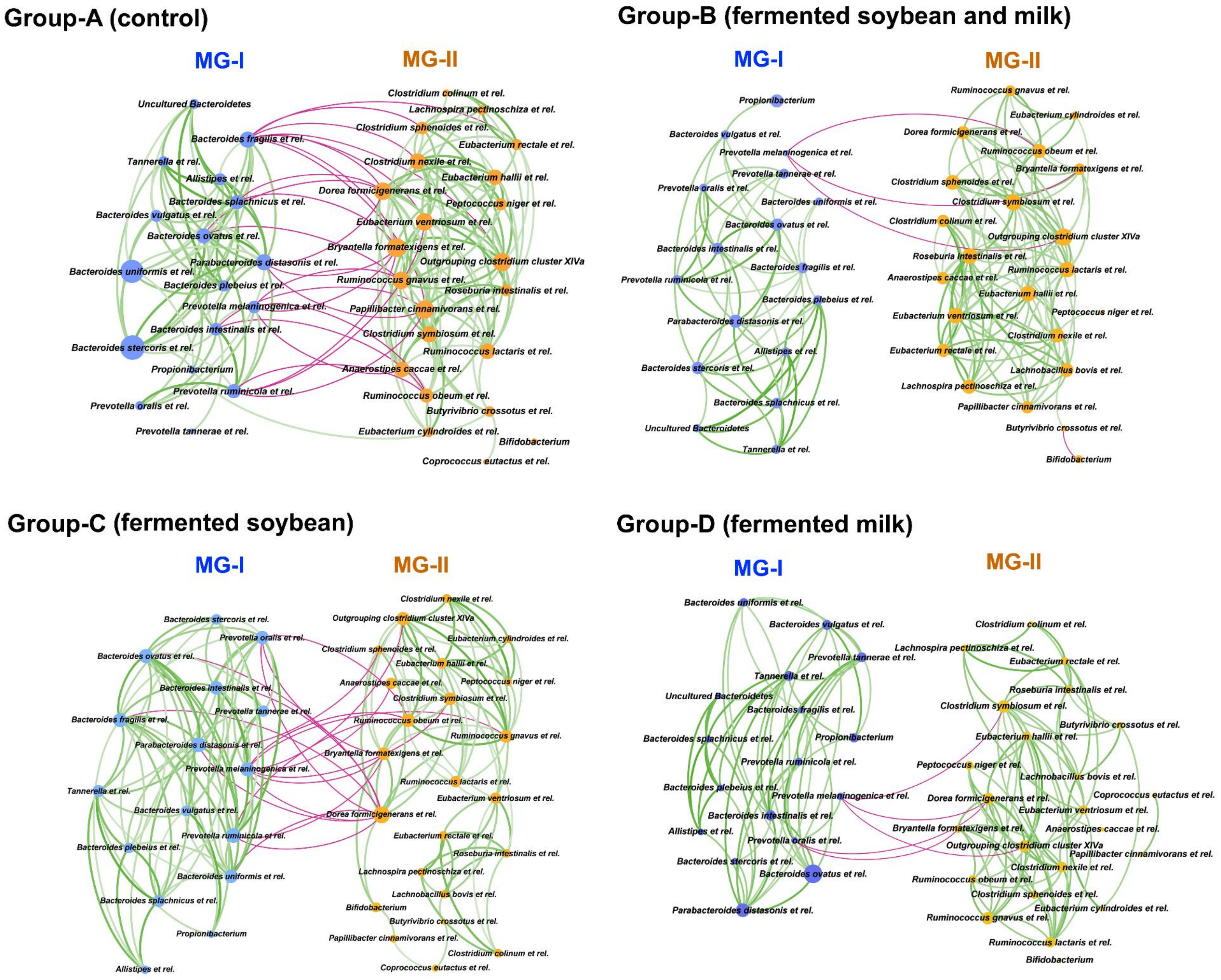

Group-A (control)
MG-I
MG-II
Uncultured Bacteroidetes
Bacteroides fragilis et rel.
Tannerella et rel.
Allistipes et rel.
Bacteroides splachnicus et rel.
Bacteroides vulgatus et rel.
Bacteroides ovatus et rel.
Parabacteroides distasonis et rel.
Bacteroides uniformis et rel.
Bacteroides plebeius et rel.
Prevotella melaninogenica et rel.
Bacteroides intestinalis et rel.
Bacteroides stercoris et rel.
Propionibacterium
Prevotella ruminicola et rel.
Prevotella oralis et rel.
Prevotella tannerae et rel.
Clostridium colinum et rel.
Lachnospira pectinoschiza et rel.
Clostridium sphenoides et rel.
Eubacterium rectale et rel.
Clostridium nexile et rel.
Eubacterium hallii et rel.
Dorea formicigenerans et rel.
Peptococcus niger et rel.
Eubacterium ventriosum et rel.
Bryantella formatexigens et rel.
Outgrouping clostridium cluster XIVa
Ruminococcus gnavus et rel.
Roseburia intestinalis et rel.
Papillibacter cinnamivorans et rel.
Clostridium symbiosum et rel.
Ruminococcus lactaris et rel.
Anaerostipes caccae et rel.
Ruminococcus obeum et rel.
Butyrivibrio crossotus et rel.
Eubacterium cylindroides et rel.
Bifidobacterium
Coprococcus eutactus et rel.
Group-B (fermented soybean and milk)
MG-I
MG-II
Propionibacterium
Bacteroides vulgatus et rel.
Prevotella melaninogenica et rel.
Prevotella tannerae et rel.
Prevotella oralis et rel.
Bacteroides uniformis et rel.
Bacteroides ovatus et rel.
Bacteroides intestinalis et rel.
Bacteroides fragilis et rel.
Prevotella ruminicola et rel.
Bacteroides plebeius et rel.
Parabacteroides distasonis et rel.
Allistipes et rel.
Bacteroides stercoris et rel.
Bacteroides splachnicus et rel.
Uncultured Bacteroidetes
Tannerella et rel.
Ruminococcus gnavus et rel.
Eubacterium cylindroides et rel.
Dorea formicigenerans et rel.
Ruminococcus obeum et rel.
Bryantella formatexigens et rel.
Clostridium sphenoides et rel.
Clostridium symbiosum et rel.
Clostridium colinum et rel.
Outgrouping clostridium cluster XIVa
Roseburia intestinalis et rel.
Ruminococcus lactaris et rel.
Anaerostipes caccae et rel.
Eubacterium hallii et rel.
Peptococcus niger et rel.
Eubacterium ventriosum et rel.
Clostridium nexile et rel.
Eubacterium rectale et rel.
Lachnobacillus bovis et rel.
Lachnospira pectinoschiza et rel.
Papillibacter cinnamivorans et rel.
Butyrivibrio crossotus et rel.
Bifidobacterium
Group-C (fermented soybean)
MG-I
MG-II
Bacteroides stercoris et rel.
Prevotella oralis et rel.
Bacteroides ovatus et rel.
Bacteroides intestinalis et rel.
Prevotella tannerae et rel.
Bacteroides fragilis et rel.
Parabacteroides distasonis et rel.
Prevotella melaninogenica et rel.
Tannerella et rel.
Bacteroides vulgatus et rel.
Prevotella ruminicola et rel.
Bacteroides plebeius et rel.
Bacteroides uniformis et rel.
Bacteroides splachnicus et rel.
Propionibacterium
Allistipes et rel.
Clostridium nexile et rel.
Outgrouping clostridium cluster XIVa
Eubacterium cylindroides et rel.
Clostridium sphenoides et rel.
Eubacterium hallii et rel.
Anaerostipes caccae et rel.
Peptococcus niger et rel.
Clostridium symbiosum et rel.
Ruminococcus obeum et rel.
Ruminococcus gnavus et rel.
Bryantella formatexigens et rel.
Ruminococcus lactaris et rel.
Eubacterium ventriosum et rel.
Dorea formicigenerans et rel.
Eubacterium rectale et rel.
Roseburia intestinalis et rel.
Lachnospira pectinoschiza et rel.
Lachnobacillus bovis et rel.
Bifidobacterium
Butyrivibrio crossotus et rel.
Papillibacter cinnamivorans et rel.
Clostridium colinum et rel.
Coprococcus eutactus et rel.
Group-D (fermented milk)
MG-I
MG-II
Bacteroides uniformis et rel.
Bacteroides vulgatus et rel.
Prevotella tannerae et rel.
Tannerella et rel.
Uncultured Bacteroidetes
Bacteroides fragilis et rel.
Bacteroides splachnicus et rel.
Propionibacterium
Prevotella ruminicola et rel.
Bacteroides plebeius et rel.
Prevotella melaninogenica et rel.
Bacteroides intestinalis et rel.
Allistipes et rel.
Prevotella oralis et rel.
Bacteroides stercoris et rel.
Bacteroides ovatus et rel.
Parabacteroides distasonis et rel.
Clostridium colinum et rel.
Lachnospira pectinoschiza et rel.
Eubacterium rectale et rel.
Roseburia intestinalis et rel.
Clostridium symbiosum et rel.
Butyrivibrio crossotus et rel.
Eubacterium hallii et rel.
Peptococcus niger et rel.
Lachnobacillus bovis et rel.
Dorea formicigenerans et rel.
Coprococcus eutactus et rel.
Eubacterium ventriosum et rel.
Bryantella formatexigens et rel.
Anaerostipes caccae et rel.
Outgrouping clostridium cluster XIVa
Papillibacter cinnamivorans et rel.
Clostridium nexile et rel.
Ruminococcus obeum et rel.
Clostridium sphenoides et rel.
Eubacterium cylindroides et rel.
Ruminococcus gnavus et rel.
Ruminococcus lactaris et rel.
Bifidobacterium

Figure S16 The difference in the co-exclusion network between *Clostridium* cluster XIVa (*Lachnospiraceae*) and *Bacteroidota* visualised in the four study groups (Group-A: not consumed *Dahi* and *Hawaijar* (n=58), Group-B: consumed *Dahi* and *Hawaijar* (n=54), Group-C: consumed *Hawaijar*, not *Dahi* (n=65), and Group-D: consumed *Dahi*, not *Hawaijar* (n=37)). The correlation network plots show the difference in the negative interaction (Spearman correlation coefficient <-0.4, $p<0.0001$) between two modularity groups of co-occurring taxa (Spearman correlation coefficient $>+0.4$, $p<0.0001$), namely modularity group-I (MG-I) with members of *Clostridium* cluster XIVa and modularity group-II (MG-II) with members of *Bacteroidota*. Source data are provided as a Source Data file. The green line indicates positive interaction, and the pinkish-red line indicates negative interaction. The colour of the nodes indicates their modularity group assignment (blue colour for MG-I and brown colour for MG-II), and the size of the node indicates the number of edges adjacent to the node.

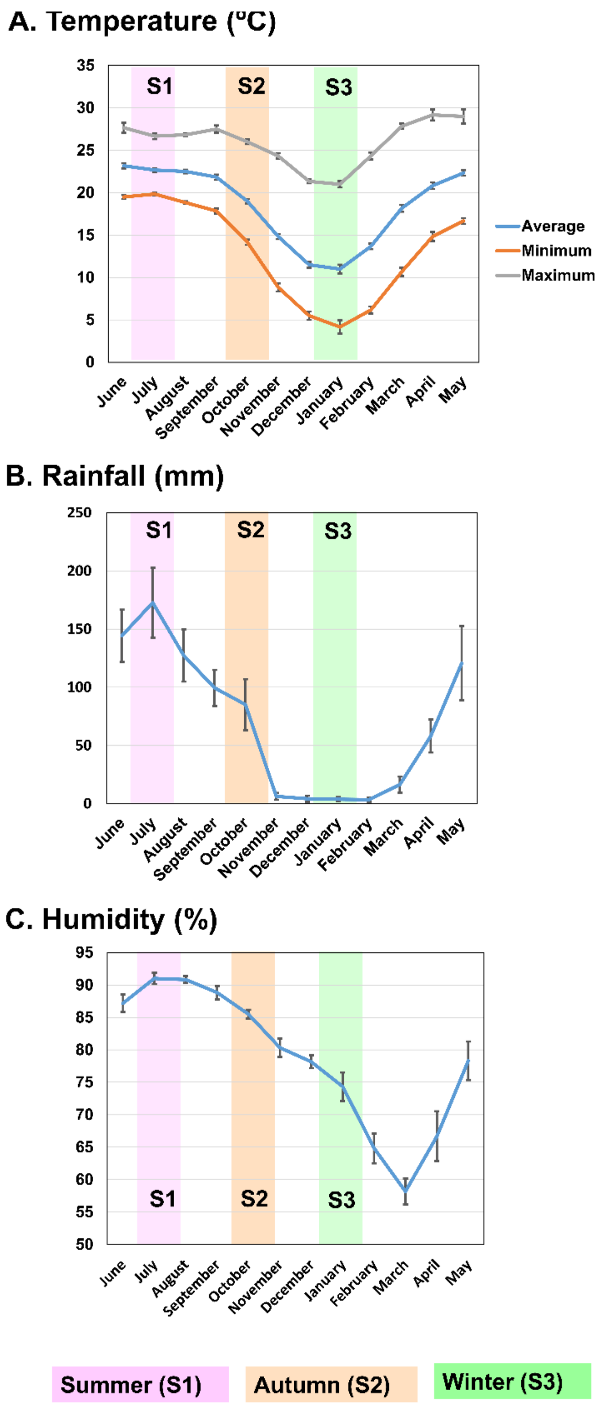

Figure S17 Seasonal changes in the temperature, rainfall and humidity (n=12) in Imphal, Manipur, India, during the three-time intervals of sample collection are visualised here. The seasonal cyclic changes plotted here are average six-year data from 2010 to 2015 (Source: https://www.worldweatheronline.com/lang/en-in/imphal-weather-averages/manipur/in.aspx). The trend line chart is visualised as mean± s.e.m, and the error bars represent s.e.m . Source data are provided as a Source Data file.

Supplementary Tables

Table S1 Metadata of the study subjects in each categorised group (Data on single subjects are available as an Excel file in Source Data)

		Group A	Group B	Group C	Group D
Number		20	21	23	14
Age (years)	< 14	1	3	2	0
	15-24	3	1	3	3
	25-34	6	5	6	3
	35-44	4	5	4	3
	45-54	4	5	5	2
	55-64	1	2	3	1
	>65	1	0	0	2
Body Mass Index (BMI)	<18.5	5	2	0	1
	18.5-25	10	13	12	4
	25-30	4	4	10	9
	>30	1	2	1	0
Sex	Male	13	10	10	7
	Female	7	11	13	7
Clan	Mangang	7	12	10	6
	Luwang	2	2	2	1
	Khuman	2	2	6	1
	Angom	1	2	1	1
	Kha-Nganba	4	2	1	1
	Moirang	4	1	3	4
Nature of Birth	Vaginal	19	21	22	14
	Caesarean	1	0	1	0
Marital status	Married	13	15	16	9
	Unmarried	7	6	7	5
Long-term Food habit	Fish-eating vegetarian	2	2	6	0
	Non-vegetarian	18	19	17	14

Table S2 The absolute abundance of gut bacterial taxa discriminating the two clusters (cluster-P: n=117 and cluster-B/R: n=97) viewed in the Indian study population

Major drivers	Taxa (genus-like)	Absolute abundance[a] (Median ± SEM, Log 10 16S rRNA gene copies/g of wet faecal sample)		Corrected p-value (two-sided Wilcoxon test, Bonferroni corrected)
		Cluster -P	Cluster-B/R	
Prevotella	*Prevotella melaninogenica* et rel.	9.93±0.11	7.33±0.41	1.07E-24
	Prevotella tannerae et rel.	8.93±0.07	8.10±0.09	3.05E-11
	Prevotella oralis et rel.	8.94±0.11	7.95±0.09	1.61E-13
	Prevotella ruminicola et rel.	8.80±0.05	8.40±0.05	1.52E-06
Bifidobacterium	*Bifidobacterium*	8.30±0.11	8.26±0.16	0.61
Clostridium cluster XIVa (*Lachnospiraceae*)	*Anaerostipes caccae* et rel.	8.35±0.05	8.21±0.07	0.05
	Bryantella formatexigens et rel.	8.73±0.14	8.62±0.19	0.30
	Ruminococcus gnavus et rel.	8.78±0.14	8.75±0.07	0.64
	Ruminococcus obeum et rel.	9.13±0.14	9.09±0.15	0.38
	Clostridium symbiosum et rel.	8.70±0.39	8.52±0.35	0.76

[a]The absolute numbers of taxa were calculated based on relative abundances multiplied by the total bacterial load determined by 16S rRNA gene counts.

Table S3 Identities of the faecal metabolites that significantly differed between the two gut bacterial clusters (*Prevotella*-driven cluster-P: n=41, compared to *Bifidobacterium*/*Ruminococcus*-driven cluster-B/R: n=30, at baseline summer) viewed in the Indian study population

Comp ID	Comp MW	Fold change[a]	Significance (p-value)[b]	Suggested IDs from ChemSpider/ mzCloud identity (score)
C488	180.07248	2.01	0.00157	Protionamide
C522	185.10516	2.43	0.00252	(2R,3S)-3-Hydroxy-8-methyl-8-azabicyclo[3.2.1]octane-2-carboxylic acid, (2S,3S)-3-Hydroxy-8-methyl-8-azabicyclo[3.2.1]octane-2-carboxylic acid, (4R,6Z)-4-Hydroxy-6-(hydroxymethyl)-1-methyl-1,3,4,8-tetrahydro-5(2H)-azocinone, 3-Methyl-N-[(3S)-2-oxotetrahydro-3-furanyl]butanamide
C594	194.09355	3.69	0.00220	Butylparaben (50.3)
C687	211.12118	2.53	0.01971	Isoprenaline, Mescaline, orciprenaline, Methoxamine, N-Ethylethanaminium salicylate, Proternol L
C708	215.17729	3.68	0.00111	NA
C878	242.01883	2.65	0.00004	1D-myo-Inositol 1,2-cyclic phosphate, 1-Deoxy-6-O-phosphono-D-threo-hexo-2,5-diulose
C1116	282.16227	3.99	0.00104	Vedaprofen, 2-Isopropyl-8,8-dimethyl-5,6,7,8-tetrahydro-3,4-phenanthrenedione, 3-Hydroxy-1-methylestra-1,3,5(10),6-tetraen-17-one, 2-(3-Methyl-2-buten-1-yl)-5-(2-phenylethyl)-1,3-benzenediol, Mestilbol (VAN), 4-[(6E)-3-Hydroxy-7-phenyl-6-hepten-1-yl]phenol, UNII:1V339IMQ38, hexaethyleneglycol

C1166	288.03166	-4.82	0.01025	Ciprofibrate, 2-Hydroxy-5-[(5-oxotetrahydro-2-furanyl)methyl]phenyl hydrogen sulfate
C1170	288.19377	4.15	0.00350	6-Hydroxypentadecanedioic acid
C1222	298.12083	2.22	0.00073	Benzyl succinate, Enterolactone, 3,4-Bis(3-hydroxybenzyl)dihydro-2(3H)-furanone, 4,4''-Tetrahydro-1H,3H-furo[3,4-c]furan-1,4-diyldiphenol, 2'',4''-dihydroxy-6''-methoxy-3'',5''-dimethylchalcone, 7-Hydroxy-5-methoxy-6,8-dimethyl-2-phenyl-2,3-dihydro-4H-chromen-4-one, 5,7-Dimethoxy-6-methyl-2-phenyl-2,3-dihydro-4H-chromen-4-one, (4Z)-4-Methyl-6-(3-methyl-1,4-dioxo-1,4-dihydro-2-naphthalenyl)-4-hexenoic acid, 2-Methyl-4-(1-piperazinyl)-10H-thieno[2,3-b][1,5]benzodiazepine, Metadoxine
C1343	324.16868	2.03	0.00104	3,4,5-Trimethoxybenzamido-N,N-diethylacetamide, 1a-[(2E)-3,7-Dimethyl-2,6-octadien-1-yl]-7a-methyl-1a,7a-dihydronaphtho[2,3-b]oxirene-2,7-dione, Tecastemizole, Citalopram, Escitalopram
C1380	330.11201	2.09	0.00033	1,4-Naphthalenedione, 2-(1-(acetyloxy)-4-methyl-3-pentenyl)-5,8-dihydroxy-, 4''-Hydroxy-5,6,7-trimethoxyflavanone, hamiltone A, samaderin A, 1,2-benzenediol, 4-[(2R,3R)-2,3-dihydro-3-(hydroxymethyl)-6-[(1E)-3-hydroxy-1-propenyl]-1,4-benzodioxin-2-yl]-, 3,9,10-Trimethoxy-6a,11a-dihydro-6H-[1]benzofuro[3,2-c]chromen-4-ol, 3'',6''-Dihydroxy-2'',4,4''-trimethoxy-chalcone, 5-Hydroxy-3-(4-hydroxybenzyl)-7,8-dimethoxy-2,3-dihydro-4H-chromen-4-one, 7-Hydroxy-3-(3-hydroxy-4-methoxybenzyl)-5-

				methoxy-2,3-dihydro-4H-chromen-4-one, 2,3,9-Trimethoxy-6a,11a-dihydro-6H-[1]benzofuro[3,2-c]chromen-4-ol, 2-(2,4-Dimethoxyphenyl)-7-hydroxy-5-methoxy-2,3-dihydro-4H-chromen-4-one, 2-(1,3-Benzodioxol-5-yl)-5,7-dimethoxy-3-chromanol, 2-Hydroxy-2-(4-hydroxyphenyl)ethyl (2E)-3-(4-hydroxy-3-methoxyphenyl)acrylate, 4-{3-(Hydroxymethyl)-6-[(1Z)-3-hydroxy-1-propen-1-yl]-2,3-dihydro-1,4-benzodioxin-2-yl}-1,2-benzenediol, 4-{3-(Hydroxymethyl)-7-[(1Z)-3-hydroxy-1-propen-1-yl]-2,3-dihydro-1,4-benzodioxin-2-yl}-1,2-benzenediol, 3-(1,2-Dihydroxyethyl)-9-methoxy-3,4,7,8-tetrahydro-2H-cyclopenta[5,6]naphtho[2,1-b]furan-5,6-dione, N''-Benzoyl-N''-tert-butyl-4-chlorbenzohydrazid
C1446	346.18574	-2.87	0.00009	NA
C1454	348.26637	2.22	0.00055	Anacardic acid (58.8)
C1649	400.04640	-2.19	0.02754	Bis(2,3-dihydroxypropyl) 2-hydroxy-1,3-propanediyl bis[hydrogen (phosphate)]
C1719	423.27562	2.96	0.00005	NA
C1753	434.34193	2.06	0.00206	3alpha,7alpha,12alpha-trihydroxy-5beta-cholestan-26-al, 3a,7a-Dihydroxycoprostanic acid, (1S,3R,5Z,7E,24R)-9,10-Secocholesta-5,7,10-triene-1,3,24-triol hydrate (1:1), (24S)-3alpha,7alpha,24-trihydroxy-5beta-cholestan-26-al, (3alpha,5beta,7alpha,8xi)-3,7-Dihydroxycholestan-26-oic acid

C1767	439.27214	2.38	6.90E-07	(2R)-1-{[(2-Aminoethoxy)(hydroxy)phosphoryl]oxy}-3-hydroxy-2-propanyl pentadecanoate, (2R)-3-{[(2-Aminoethoxy)(hydroxy)phosphoryl]oxy}-2-hydroxypropyl pentadecanoate
C1806	453.28635	2.00	7.51E-06	1-hexadecanoyl-sn-glycero-3-phosphoethanolamine, 2-hexadecanoyl-sn-glycero-3-phosphoethanolamine
C1871	472.23427	-2.29	0.01870	8-[(3R)-3-Amino-1-piperidinyl]-7-(2-butyn-1-yl)-3-methyl-1-[(4-methyl-2-quinazolinyl)methyl]-3,7-dihydro-1H-purine-2,6-dione
C1943	501.29072	3.92	0.04418	Avasimibe, Fexofenadine, (3R,5bS,7aS,13bS,13cR,15aS)-5b-hydroxy-2,2,13b,13c-tetramethyl-9-(2-methylbut-3-en-2-yl)-2,3,5b,6,7,7a,8,13,13b,13c,14,15-dodecahydro-4H-3,15a-epoxy[1]benzoxepino[6",7":6,7]indeno[1,2-b]indol-4-one, (2R)-3-{[(2-Aminoethoxy)(hydroxy)phosphoryl]oxy}-2-hydroxypropyl (5Z,8Z,11Z,14Z)-5,8,11,14-icosatetraenoate, 2-arachidonoyl-sn-glycero-3-phosphoethanolamine zwitterion, (2R)-1-{[(2-Aminoethoxy)(hydroxy)phosphoryl]oxy}-3-hydroxy-2-propanyl (8Z,11Z,14Z,17Z)-8,11,14,17-icosatetraenoate, (2R)-3-{[(2-Aminoethoxy)(hydroxy)phosphoryl]oxy}-2-hydroxypropyl (8Z,11Z,14Z,17Z)-8,11,14,17-icosatetraenoate
C2006	531.13795	3.71	0.00129	NA
C2138	658.46138	4.92	0.04667	NA

[a]Log2 fold change in *Prevotella*-driven cluster-P compared to *Bifidobacterium*/*Ruminococcus*-driven cluster-B/R, the negative value indicates a higher relative abundance of metabolites in cluster-B/R.

[b] Two-sided Wilcoxon test, BH corrected p-value

Table S4 Bacterial community structure of *Hawaijar* (fermented soybean) analysed by Illumina-MiSeq amplicon sequencing

NCBI-SRA Accession Study: PRJNA1191989		SRX26913732	SRX26913734	SRX26913737	SRX26913739	SRX26913723
Phylum	Firmicutes	96.47	97.52	88.49	98.70	82.45
	Proteobacteria	3.45	2.02	11.47	1.30	16.82
	Cyanobacteria	0.00	0.00	0.04	0.00	0.73
	Deinococcota	0.00	0.43	0.00	0.00	0.00
	Actinobacteriota	0.08	0.03	0.00	0.00	0.00
Family	*Bacillaceae*	88.65	87.82	38.11	93.36	10.41
	Enterococcaceae	3.43	4.30	10.80	2.51	20.02
	Leuconostocaceae	0.15	0.18	15.12	0.17	3.29
	Lactobacillaceae	0.65	0.42	0.50	0.64	15.91
	Staphylococcaceae	0.44	2.61	0.88	0.25	8.97
	Carnobacteriaceae	1.20	1.24	0.71	1.34	8.47
	Planococcaceae	0.13	0.09	10.75	0.00	0.41
	Vagococcaceae	0.35	0.24	0.36	0.00	9.74
	Moraxellaceae	1.02	0.00	4.86	0.00	4.79
	Streptococcaceae	0.11	0.08	0.11	0.00	5.89
	Clostridiaceae	0.12	0.07	5.97	0.00	0.00
	Aerococcaceae	0.18	0.14	4.49	0.31	0.00
	Others	3.57	2.80	7.34	1.41	12.10
Genus	*Bacillus*	88.56	87.74	38.11	93.36	9.22
	Enterococcus	3.43	4.30	10.80	2.51	13.29
	Ignatzschineria	0.23	0.18	0.25	0.17	3.61
	Lactobacillus	0.65	0.42	0.50	0.64	15.91
	Weissella	0.00	0.00	14.48	0.00	2.26
	Atopostipes	1.20	1.24	0.71	1.34	8.47
	Staphylococcus	0.44	2.61	0.88	0.25	8.41
	Vagococcus	0.35	0.24	0.36	0.00	9.74
	Kurthia	0.13	0.09	9.93	0.00	0.26
	Tetragenococcus	0.00	0.00	0.00	0.00	6.73
	Acinetobacter	1.02	0.00	4.86	0.00	0.32
	Streptococcus	0.00	0.00	0.11	0.00	4.41
	Psychrobacter	0.00	0.00	0.00	0.00	4.47
	Paraclostridium	0.00	0.00	0.00	0.12	2.13
	Proteus	0.00	0.00	3.92	0.00	0.00
	Halomonas	1.51	1.15	0.53	0.54	0.00
	Acetobacter	0.19	0.00	0.61	0.20	2.60
	Globicatella	0.00	0.00	3.38	0.00	0.00
	Leuconostoc	0.15	0.18	0.65	0.17	1.03
	Others	2.13	1.84	9.92	0.68	7.14

Table S5 Bacterial community structure of *Dahi* (fermented milk) analysed by Illumina-MiSeq amplicon sequencing

NCBI-SRA Accession Study: PRJNA1191989		SRX26913726	SRX26913728	SRX26913729	SRX26913730	SRX26913731
Phylum	Firmicutes	85.37	91.94	61.51	78.82	80.32
	Proteobacteria	12.87	8.06	38.49	21.18	18.04
	Cyanobacteria	1.76	0.00	0.00	0.00	1.65
	Deinococcota	0.00	0.00	0.00	0.00	0.00
	Actinobacteriota	0.00	0.00	0.00	0.00	0.00
Family	*Streptococcaceae*	41.46	71.30	12.52	59.06	16.27
	Acetobacteraceae	4.01	6.72	34.40	19.24	8.09
	Leuconostocaceae	4.67	18.62	19.12	14.81	4.52
	Enterococcaceae	9.99	1.29	28.69	4.05	14.62
	Lactobacillaceae	14.26	0.22	0.64	0.22	22.17
	Staphylococcaceae	6.01	0.08	0.16	0.00	11.47
	Carnobacteriaceae	7.11	0.07	0.00	0.14	7.77
	Wohlfahrtiimonadaceae	3.22	0.00	0.00	0.00	3.70
	Bacillaceae	1.89	0.22	0.25	0.37	2.65
	Vagococcaceae	1.80	0.00	0.00	0.00	3.51
	Peptostreptococcaceae	2.32	0.13	0.12	0.13	1.58
	Others	3.25	1.34	4.09	1.98	3.66
Genus	*Lactococcus*	37.14	71.21	12.29	58.99	8.50
	Acetobacter	4.01	6.72	34.03	19.14	8.09
	Leuconostoc	2.21	18.62	19.12	14.81	1.74
	Enterococcus	5.72	1.29	28.69	4.05	7.04
	Lactobacillus	14.26	0.22	0.64	0.22	22.17
	Staphylococcus	6.01	0.08	0.16	0.00	11.15
	Atopostipes	7.11	0.07	0.00	0.14	7.77
	Streptococcus	4.33	0.10	0.24	0.08	7.77
	Tetragenococcus	4.27	0.00	0.00	0.00	7.58
	Ignatzschineria	3.22	0.00	0.00	0.00	3.70
	Vagococcus	1.80	0.00	0.00	0.00	3.51
	Weissella	2.47	0.00	0.00	0.00	2.78
	Bacillus	1.28	0.11	0.25	0.37	1.90
	Hafnia	0.00	0.17	2.88	0.66	0.00
	Paraclostridium	0.99	0.13	0.12	0.13	1.58
	Klebsiella	0.00	0.00	0.00	0.00	2.46
	Pseudomonas	0.06	0.84	0.11	0.65	0.00
	Acinetobacter	0.64	0.11	0.09	0.31	0.51
	Geobacillus	0.61	0.00	0.00	0.00	0.76
	Others	3.89	0.32	1.38	0.46	1.01

Table S6 Benjamini-Hochberg method adjusted p-values for each diet with respect to the control Group-A for the taxa with significant diet effects (Tukey post hoc test of the linear mixed-effects model results, FDR<0.25, absolute fold change>2 in HITChip signal).

	**Group-B**	**Group-C**	**Group-D**
Prevotella tannerae et rel.	0.93	0.02	0
Tannerella et rel.	0.65	0	0
Bacteroides splachnicus et rel.	0.46	0.01	0
Catenibacterium mitsuokai et rel.	0.23	0.98	0
Parabacteroides distasonis et rel.	0.71	0.04	0

Table S7 Benjamini-Hochberg method adjusted p-values for each season with respect to the control season Autumn for the taxa with significant season effects (Tukey post hoc test of the linear mixed-effects model results, FDR<0.25, absolute fold change>2 in HITChip signal).

	**Summer**	**Winter**
Bacteroides plebeius et rel.	0	0.93
Weissella et rel.	0.14	0
Bacteroides stercoris et rel.	0	0.64
Allistipes et rel.	0	0.53
Bacteroides uniformis et rel.	0	0.73
Anaerofustis	0.04	0.02
Bacteroides splachnicus et rel.	0	0.61
Prevotella tannerae et rel.	0	0.81
Bacteroides vulgatus et rel.	0	0.6
Prevotellaoralis et rel.	0	0.96
Parabacteroides distasonis et rel.	0	0.46
Bacteroides intestinalis et rel.	0	0.56
Bacteroides ovatus et rel.	0	0.45
*Bacteroides fragilis*et rel.	0.01	0.35
Prevotella ruminicola et rel.	0	0.56
Tannerella et rel.	0	0.56
Eggerthellalenta et rel.	0.01	0.36
*Lactobacillus gasseri*et rel.	0	0.65
Serratia	0.19	0.03
Streptococcus bovis et rel.	0.24	0.06
Uncultured Bacteroidota	0	0.99
Sutterella wadsworthia et rel.	0.19	0.11
Aerococcus	0.06	0.38
Granulicatella	0.14	0.22

Table S8 List of primers used for taxa-specific quantification of gut bacteria by qPCR analysis

SI. No	Target group	Primer	Sequence (5'-3')	Strain used as Standard	Annealing temperature	References
1	Total bacteria	Prok-1492R<br>Bact-1369R	GGWTACCTTGTTACGACTT<br>CGGTGAATACGTTCYCGG	*Escherichia coli* DH5α	56.0 ºC	4
2	Bacteroidota	Bact F<br>Bact R	GGARCATGTGGTTTAATTCGATGAT<br>AGCTGACGACAACCATGCAG	*Bacteroides thetaiotaomicron* DSM 2079	60.0 ºC	5
3	Bacillota	Firm F<br>Firm R	GGAGYATGTGGTTTAATTCGAAGCA<br>AGCTGACGACAACCATGCAC	*Ruminococcus gnavus* ATCC 29149	48.0 ºC	5
4	*Methanobrevibacter smithii*	Smit -16S-740F<br>Smit 16S 862R	CCGGGTATCTAATCCGGTTC<br>CTCCCAGGGTAGAGGTGAAA	*Methanobrevibacter smithii* DSM 274	57.2 ºC	6
6	Lactic acid bacteria	Lab 1 for<br>Lab 0677 rev	AGCAGTAGGGAATCTTCCA<br>CACGGCTACACATGGAG	*Ligilactobacillus plantarum* WCFS1	60.0 ºC	7
7	*Bacillus subtilis* phylogeny group	Bacil_for<br>Bacil_ rev	TGGCTTCGGCTACCACTTAC<br>GTTGCTCCGTCAGACTTTCG	*Bacillus subtilis* ATCC6051	61.0 ºC	8
8	*Prevotella*	Prev F<br>Prev R	GGTTCTGAGAGGAAGGTCCCC<br>GAGTTTGATCCTGGCTCAG	*Prevotella copri* DSM-18205	58.5 ºC	9
9	*Bifidobacterium*	Bifi F<br>Bifi R	TCGCGTCYGGTGTGAAAG<br>CCACATCCAGCRTCCAC	*Bifidobacterium longum* subsp. *longum* DSM 20219	58.0 ºC	10
10	*Bacteroides*	Bact F<br>Bact R	GTCAGTTGTGAAAGTTTGC<br>CAATCGGAGTTCTTCGTG	*Bacteroides fragilis*	56.0 ºC	11

Supplementary References:

1. Keisam, S., Romi, W., Ahmed, G. & Jeyaram, K. Quantifying the biases in metagenome mining for realistic assessment of microbial ecology of naturally fermented foods. *Sci. Rep.* **6**, 34155 (2016).
2. Romi, W., Ahmed, G. & Jeyaram, K. Three-phase succession of autochthonous lactic acid bacteria to reach a stable ecosystem within 7 days of natural bamboo shoot fermentation as revealed by different molecular approaches. *Mol. Ecol.* **24**, 3372-3389 (2015).
3. Caporaso, J.G. *et al*. Ultra-high-throughput microbial community analysis on the Illumina HiSeq and MiSeq platforms. *ISME J.* **6**.1621-1624 (2012).
4. Suzuki, M. T., Taylor, L. T. & DeLong, E. F. Quantitative analysis of small-subunit rRNA genes in mixed microbial populations via 5'-nuclease assays. *Appl. Environ. Microbiol.***66,** 4605-4614 (2000).
5. Guo, X. *et al.* Development of a real-time PCR method for Firmicutes and Bacteroidetes in faeces and its application to quantify intestinal population of obese and lean pigs. *Lett. Appl. Microbiol.* **47,** 367-373 (2008).
6. Dridi, B., Henry, M., El Khéchine, A., Raoult, D. & Drancourt, M. High prevalence of *Methanobrevibacter smithii* and *Methanosphaera stadtmanae* detected in the human gut using an improved DNA detection protocol. *PLoS One***4,** e7063 (2009).
7. Wieschebrock, M., Seitter, M. & Hertel, C. Quantitative detection of lactic acid bacteria in dried sourdoughs using real-time PCR. *Eur. Food Res. Technol.***7,** 624 (2011).
8. Heilig, H. G. H. J. *et al.* Molecular diversity of Lactobacillus spp. and other lactic acid bacteria in the human intestine as determined by specific amplification of 16S ribosomal DNA. *Appl. Environ. Microbiol.***68,** 114-123(2002).
9. Bekele, A. Z., Koike, S. & Kobayashi, Y. Genetic diversity and diet specificity of ruminal *Prevotella* revealed by 16S rRNA gene-based analysis. *FEMS Microbiol. Lett.***305,** 49-57

(2010).

10. Rinttilä, T., Kassinen, A., Malinen, E., Krogius, L. & Palva, A. Development of an extensive set of 16S rDNA-targeted primers for quantification of pathogenic and indigenous bacteria in faecal samples by real-time PCR. *J. Appl. Microbiol.* **97,** 1166-1177 (2004).

11. Ahmed, S. *et al.* Mucosa-associated bacterial diversity in relation to human terminal ileum and colonic biopsy samples. *Appl. Environ. Microbiol.* **73,** 7435-7442 (2007).
