# Supplementary material for: Fermented foods affect the seasonal stability of gut bacteria in an Indian rural population
Source: Nat Commun. 2025 Jan 17;16:771. doi: 10.1038/s41467-025-56014-6 (PMC11748640; doi:10.1038/s41467-025-56014-6)
Supplement: Supplementary file 2 — Description of Additional Supplementary Files [file 41467_2025_56014_MOESM2_ESM.pdf]

Description of Additional Supplementary Files

File Name: Supplementary Data 1

Description: Sheet 1: Differences in the long-term habitual diets and lifestyles of 78 subjects recruited in this study.

Sheet 2: Differences in the 48-hour diet recall data during seasonal sampling and associated bacterial community clusters in individual subjects in each categorized study group.
