# Supplementary material for: Fermented foods affect the seasonal stability of gut bacteria in an Indian rural population
Source: Nat Commun. 2025 Jan 17;16:771. doi: 10.1038/s41467-025-56014-6 (PMC11748640; doi:10.1038/s41467-025-56014-6)
Supplement: Supplementary file 4 — Reporting Summary [file 41467_2025_56014_MOESM4_ESM.pdf]

nature portfolio

Corresponding author(s): Kumaraswamy Jeyaram

Last updated by author(s): Dec 31, 2024

Reporting Summary

Nature Portfolio wishes to improve the reproducibility of the work that we publish. This form provides structure for consistency and transparency in reporting. For further information on Nature Portfolio policies, see our Editorial Policies and the Editorial Policy Checklist.

Statistics

For all statistical analyses, confirm that the following items are present in the figure legend, table legend, main text, or Methods section.

n/a	Confirmed	
☐	☒	The exact sample size (*n*) for each experimental group/condition, given as a discrete number and unit of measurement
☐	☒	A statement on whether measurements were taken from distinct samples or whether the same sample was measured repeatedly
☐	☒	The statistical test(s) used AND whether they are one- or two-sided<br>*Only common tests should be described solely by name; describe more complex techniques in the Methods section.*
☐	☒	A description of all covariates tested
☐	☒	A description of any assumptions or corrections, such as tests of normality and adjustment for multiple comparisons
☐	☒	A full description of the statistical parameters including central tendency (e.g. means) or other basic estimates (e.g. regression coefficient) AND variation (e.g. standard deviation) or associated estimates of uncertainty (e.g. confidence intervals)
☐	☒	For null hypothesis testing, the test statistic (e.g. *F*, *t*, *r*) with confidence intervals, effect sizes, degrees of freedom and *P* value noted<br>*Give P values as exact values whenever suitable.*
☒	☐	For Bayesian analysis, information on the choice of priors and Markov chain Monte Carlo settings
☒	☐	For hierarchical and complex designs, identification of the appropriate level for tests and full reporting of outcomes
☐	☒	Estimates of effect sizes (e.g. Cohen's *d*, Pearson's *r*), indicating how they were calculated

Our web collection on statistics for biologists contains articles on many of the points above.

Software and code

Policy information about availability of computer code

Data collection	The human gut microbiota profile (HITChip and taxa specific qPCR assays) and fecal metabolite profile (LC-HRMS and HPLC) of Indian study population (n=78, three time point samples), who differed in the intake of fermented foods categorized into four diet groups (n~20 for each group). For comparison, a healthy European population (n=78 subjects, from across Europe) matched for sex, BMI, and age was randomly selected from the HITChip database of the Laboratory of Microbiology, Wageningen University & Research, The Netherlands. The microbiota profile of two fermented foods (Hawaijar and Dhahi) samples (16S rRNA amplicon sequencing, n=10 and qPCR assay,n=20).
Data analysis	Agilent miarray scanner and feature extraction software, version 9.5 (http://www.agilent.com). Microarray data normalization, baseline correction and further analysis were performed in a custom-designed MySQL database management system (http://www.mysql.com/).<br>The MS and MS/MS spectra were acquired with a Dionex Ultimate 3000 ultrahigh performance L-Exactive Orbitrap (Thermo Fisher Scientific) (C-CAMP MS Facility, Bengaluru). SIEVE 2.2 for data extraction, and HMDB/KEGG database for compound identification, MS/MS scans against entries in the spectral database mzCloud.<br>High-performance liquid chromatography (Accela, Thermo).<br>qPCR assays in Biorad CFX384 (Biorad, USA) and ABI 7500 instrument (Life Technologies, USA).<br>Multivariate analysis using PAST v3.2266 and Canoco software v4.52 (Wageningen University, The Netherlands).<br>Boxplot using BoxPlotR (http://shiny.chemgrid.org/boxplotr/) and violin plots using PAST 3.22<br>Co-occurrence network analysis 'Gephi' (Version 0.8.2-beta) (https://gephi.org/) ; Microsoft Excel 2010.<br>Network analysis, Biomodality analysis, Random Forest analysis and Wilcoxon test using custom R scripts are available via Zenodo (https://doi.org/10.5281/zenodo.14369940) under an MIT license.

For manuscripts utilizing custom algorithms or software that are central to the research but not yet described in published literature, software must be made available to editors and reviewers. We strongly encourage code deposition in a community repository (e.g. GitHub). See the Nature Portfolio guidelines for submitting code & software for further information.

Data

Policy information about availability of data

All manuscripts must include a data availability statement. This statement should provide the following information, where applicable:

- Accession codes, unique identifiers, or web links for publicly available datasets
- A description of any restrictions on data availability
- For clinical datasets or third party data, please ensure that the statement adheres to our policy

Data sets used in this study, including the probe-level Human Intestinal Tract phylogenetic microarray (HITChip) data, the associated sample metadata and the derived higher-level genus and phylum level taxonomic abundance tables, qPCR data on absolute abundance and chemical profiling data are available with a permanent DOI via Zenodo (https://doi.org/10.5281/zenodo.14424024). The data is part of the HITChip Atlas (with project title "CREST STUDY"), maintained by the Laboratory of Microbiology, Wageningen University & Research. Source data for Figures are provided and described single Excel file in this paper. HPLC and LC-HRMS data are processed and visualised as Figures and Tables, and data are available in Source Data and Supplementary Information. The individual-level metadata on the diet and lifestyle habits of individuals are available in Supplementary Data. The microbiota of fermented foods data of 16S rRNA amplicon sequencing data are available in the NCBI-SRA (aaccession number: PRJNA1191989) and Other processed data generated in this study are provided in the Supplementary Information. Custom R scripts used for this study are available via Zenodo (https://doi.org/10.5281/zenodo.14369940) under an MIT license.

Research involving human participants, their data, or biological material

Policy information about studies with human participants or human data. See also policy information about sex, gender (identity/presentation), and sexual orientation and race, ethnicity and racism.

Reporting on sex and gender	The Indian study population comprised 78 subjects with 40 males (51.3 %) and 38 females (48.7 %). Self-reported biological sex was used and findings apply to both sexes. All participants signed an informed consent. A set of European individuals used for comparison (n=78; data from the HITChip database) also matched for gender.
Reporting on race, ethnicity, or other socially relevant groupings	A survey was conducted over thousands of people in a genetically and culturally homogenous population (Meitei community, Mongoloid race) across 25 sq. km areas of Imphal valley, Manipur, India. The study population belonged to the Mangang, Luwang, Khuman, Khaba-Nganba and Moirang clans of the Meitei community. These clans are strictly exogamous, whereas the overall Meitei community is endogamous with a similar food culture. Redundancy analysis by correlating metadata showed that different clans did not influence the study.
Population characteristics	The target population Meitei community of Manipur state (India) was endogamous and had similar dietary and lifestyle habits. Though they are traditionally a fish-eating vegetarian, the study population was mostly non-vegetarian (~84%), and only 16% of the subjects were fish-eating vegetarians. The study groups were balanced in age, sex, body mass index (BMI), nature of birth, dietary and lifestyle habits. They consume two meals per day with rice as a staple food. Fish, chicken and duck were the common meat items consumed by them. The study population mostly drink the government-processed tap water supply in the region. All consume tea as a regular beverage, and only 16% prefered coffee. Additional lifestyle habits of chewing areca nuts with betel leaf and occasional alcohol drinking were recorded in nearly 41% and 20%, respectively, in the subjects. The selected subjects were balanced by sex ratio, age (15-64 years) and BMI (18.5-30.0) in the categorized groups (Information included as a supplementary Table S1). All the subjects (except 2) were born vaginally and received breastfeeding in childhood. The reported staple foods were rice, pulses, seasonal vegetables and mushrooms. Among the seasonal vegetables, mustard leaves, cabbage and cauliflower were consumed mostly in the winter season. Two days' diet recall showed the consumption pattern of mustard leaves (2%, 27%,45%), cabbage (3%, 6%, 37%) and cauliflower (0%, 1%,1 6%) during different seasons (summer, autumn, winter) respectively. Among the unique vegetables consumed here, Hibiscus sabdariffa leaf was consumed mostly in summer (~20%) and Parkia speciosa pods during winter (~6%).
Recruitment	A survey was conducted over thousands of people in a genetically and culturally homogenous population of Imphal Valley regarding the frequency and quantity of fermented foods consumption. From the survey, four study groups (Group-A, Group-B, Group-C and Group-D) were identified based on the self-reported long-term dietary habit of consuming fermented foods. Based on the difference in the intake of fermented foods categorised into four diet groups, Group-A, never consumed two fermented foods (Dahi and Hawaijar) at least for the last ten years, was considered as control; Group-B is consuming both the fermented foods (Dahi and Hawaijar), Group-C is consuming Hawaijar, not Dahi, and Group-D is consuming Dahi, not consuming Hawaijar. The selection was made with the eligibility criteria of good general health, normal bowel frequency, free from any gastrointestinal diseases and other diseases, not taken antibiotic within six months before sampling. With the above eligibility criteria, about 20 subjects in each categorised study groups were randomly targeted. The study groups were balanced in age, sex, body mass index (BMI), nature of birth, dietary and lifestyle habits. Among the 85 subjects recruited, faecal samples from 78 healthy subjects were collected and analysed for the study. Temporal sampling over three different seasons, hot-humid summer (S1), autumn (S2) and cold-dry winter (S3), were collected from the study population.
Ethics oversight	The Institutional Ethical Committee (IEC) of the Institute of Bioresources and Sustainable Development, Imphal, India (approval number IBSD/IEC/2018/003) and the Department of Biotechnology, Government of India approved the study protocol. Furthermore, we obtained informed consent from all the participants and followed the guidelines of the Indian Council of Medical Research (ICMR) (www.icmr.nic.in/ethics_SOP.pdf). Written informed consent was obtained from all the participants, for the children from the children's parents or legal guardians. Samples were collected on a voluntary basis, and no compensation was provided for the participants.

Note that full information on the approval of the study protocol must also be provided in the manuscript.

Field-specific reporting

Please select the one below that is the best fit for your research. If you are not sure, read the appropriate sections before making your selection.

☒ Life sciences ☐ Behavioural & social sciences ☐ Ecological, evolutionary & environmental sciences

For a reference copy of the document with all sections, see nature.com/documents/nr-reporting-summary-flat.pdf

Life sciences study design

All studies must disclose on these points even when the disclosure is negative.

Sample size	The Post-hoc power analysis with a power of >0.85, https://homepage.univie.ac.at/robin.ristl/samplesize.php?test=ttest) was used for the sample size calculation. Our survey identified 78 healthy subjects, who differ in the intake of fermented foods categorised into four diet groups. Temporal sampling (faecal samples) over three different seasons, hot-humid summer (S1), autumn (S2) and cold-dry winter (S3), were collected from the study population. A total of n=214 samples were analysed for the Indian study population.<br>To compare the gut microbiota observed in the Indian study population, a healthy European population (n=78 subjects, from across Europe) matched for sex, BMI, and age was randomly selected from the HITChip database.<br>The final dataset included 214 samples from 78 Indian subjects and 78 samples from European subjects.<br>Consent has been obtained from the individual subjects.
Data exclusions	Among the 85 subjects targeted, seven were dropped out (three declined during the sample collection and, four experienced health issues after the first sampling due to their health condition, medication/antibiotic intake or deviation from the eligibility criteria. Seven seasonal samples were missed due to the subject's travel during the collection time. The subjects who took antibiotics during the sampling were dropped from the study groups.
Replication	HITChip, a phylogenic microarray analysis of three seasonal faecal samples from Indian subjects (214 samples) was performed with duplicates. HPLC and qPCR analysis was performed in triplicate. All replicates were successful.
Randomization	The subjects of four diet groups were randomly selected from the list of subjects qualified with the selection criteria.
Blinding	The faecal sample DNA/ metabolite extraction was kept blind without knowing the diet or season groups in the study.

Reporting for specific materials, systems and methods

We require information from authors about some types of materials, experimental systems and methods used in many studies. Here, indicate whether each material, system or method listed is relevant to your study. If you are not sure if a list item applies to your research, read the appropriate section before selecting a response.

Materials & experimental systems

n/a	Involved in the study	
☒	☐	Antibodies
☒	☐	Eukaryotic cell lines
☒	☐	Palaeontology and archaeology
☒	☐	Animals and other organisms
☒	☐	Clinical data
☒	☐	Dual use research of concern
☒	☐	Plants

Methods

n/a	Involved in the study	
☒	☐	ChIP-seq
☒	☐	Flow cytometry
☒	☐	MRI-based neuroimaging

Plants

Seed stocks	*Report on the source of all seed stocks or other plant material used. If applicable, state the seed stock centre and catalogue number. If plant specimens were collected from the field, describe the collection location, date and sampling procedures.*
Novel plant genotypes	*Describe the methods by which all novel plant genotypes were produced. This includes those generated by transgenic approaches, gene editing, chemical/radiation-based mutagenesis and hybridization. For transgenic lines, describe the transformation method, the number of independent lines analyzed and the generation upon which experiments were performed. For gene-edited lines, describe the editor used, the endogenous sequence targeted for editing, the targeting guide RNA sequence (if applicable) and how the editor was applied.*
Authentication	*Describe any authentication procedures for each seed stock used or novel genotype generated. Describe any experiments used to assess the effect of a mutation and, where applicable, how potential secondary effects (e.g. second site T-DNA insertions, mosiacism, off-target gene editing) were examined.*
