# Supplementary material for: Fermented foods affect the seasonal stability of gut bacteria in an Indian rural population
Source: Nat Commun. 2025 Jan 17;16:771. doi: 10.1038/s41467-025-56014-6 (PMC11748640; doi:10.1038/s41467-025-56014-6)
Supplement: Supplementary file 5 — The STORMS checklist [file 41467_2025_56014_MOESM5_ESM.docx]

**The STORMS checklist**

**Fermented foods affect the seasonal stability of gut bacteria in an Indian rural population (NCOMMS-24-27196B)**

Kumaraswamy Jeyaram, corresponding author jeyaram.ibsd@nic.in

STORMS checklist from https://stormsmicrobiome.org

| Number | Item | Recommendation | Item Source | Additional Guidance | Yes/No/NA | Comments or location in manuscript |
| --- | --- | --- | --- | --- | --- | --- |
| **Abstract** | | | | | | |
| 1.0 | Structured or Unstructured Abstract | Abstract should include information on background, methods, results, and conclusions in structured or unstructured format. | STORMS |  | Yes | Page 2 |
| 1.1 | Study Design | State study design in abstract. | STORMS | See 3.0 for additional information on study design. | Yes | Abstract, Page 2  Results, Page 6  Method, Page 23 |
| 1.2 | Sequencing methods | State the strategy used for metagenomic classification. | STORMS | For example, targeted 16S by qPCR or sequencing, shotgun metagenomics, metatranscriptomics, etc. | Yes | Microarray (HITChip), Illumina-MiSeq sequencing and qPCR assay used, Result, Page 6;  Method, Pages 23- 29 |
| 1.3 | Specimens | Describe body site(s) studied. | STORMS |  | Yes | Abstract, Introduction, Results, Discussion, and Methods |
| **Introduction** | | | | | | |
| 2.0 | Background and Rationale | Summarize the underlying background, scientific evidence, or theory driving the current hypothesis as well as the study objectives. | STORMS |  | Yes | Abstract, Page 2  Introduction, Page 3-6 |
| 2.1 | Hypotheses | State the pre-specified hypothesis. If the study is exploratory, state any pre-specified study objectives. | STORMS |  | Yes | Introduction, Page 6 |
| **Methods** | | | | | | |
| 3.0 | Study Design | Describe the study design. | STORMS | [Observational (Case-Control, Cohort, Cross-sectional survey, etc.) or Experimental (Randomized controlled trial, Non-randomized controlled trial, etc.). For a brief description of common study designs see: DOI: 10.11613/BM.2014.022](https://doi.org/10.1592/phco.30.10.973)  [If applicable, describe any blinding (e.g. single or double-blinding) used in the course of the study.](https://doi.org/10.1592/phco.30.10.973) | Yes | Methods, Pages 23-25  Results, Page 6 |
| 3.1 | Participants | State what the population of interest is, and the method by which participants are sampled from that population. Include relevant information on physiological state of the subjects or stage in the life history of disease under study when participants were sampled. | STORMS | Examples of the population of interest could be: adults with no chronic health conditions, adults with type II diabetes, newborns, etc. This is the total population to whom the study is hoped to be generalizable to. The sampling method describes how potential participants were selected from that population.  If the participants are from a substudy of a larger study, provide a brief description of that study and cite that study.  Clearly state how cases and controls are defined.  An example of relevant physiological state might be pre/post menopausal for a vaginal microbiome study; examples of stage in the life history of disease could be whether specimens were collected during active or dormant disease, or before or after treatment. | Yes | Method, Page 23-24.  Supplementary Table S1, Page 30.  Supplementary Methodology and Results, Pages 1-3, |
| 3.2 | Geographic location | State the geographic region(s) where participants were sampled from. | MIxS: geographic location (country and/or sea,region) | Geographic coordinates can be reported to prevent potential ambiguities if necessary. | Yes | Introduction, page 5  Methods, page 24 |
| 3.3 | Relevant Dates | State the start and end dates for recruitment, follow-up, and data collection. | STORMS | Recruitment is the period in which participants are recruited for the study. In longitudinal studies, follow-up is the date range in which participants are asked to complete a specific assessment. Finally, data collection is the total period in which data is being collected from participants including during initial recruitment through all follow-ups. | Yes | The participants were recruited from 7/3/2011 to 1/10/2012, and the details are available in the HITChip Atlas as project title “CREST STUDY” maintained by the Laboratory of Microbiology, Wageningen University & Research. HITChip data files are available via Zenodo (https://doi.org/10.5281/zenodo.14369940) under an MIT license. Data availability, Page 35. |
| 3.4 | Eligibility criteria | List any criteria for inclusion and exclusion of recruited participants. | Modified STROBE | Among potential recruited participants, how were some chosen and others not? This could include criteria such as sex, diet, age, health status, or BMI.  If there is a primary and validation sample, describe inclusion/exclusion criteria for each. | Yes | Supplementary Methodology and Results, Subject Recruitment details, Page 1  Method, Page 23-24  Results, Page 6 |
| 3.5 | Antibiotics Usage | List what is known about antibiotics usage before or during sample collection. | STORMS | If participants were excluded due to current or recent antibiotics usage, state this here.  Other factors (e.g. proton pump inhibitors, probiotics, etc.) that may influence the microbiome should also be described as well. | Yes | Supplementary Methodology and Results, Page 1.  Method, Page 24  Seven subjects were discontinued after the first sampling due to antibiotic intake or deviation from the eligibility criteria.  The inclusion criteria of no antibiotic use for a minimum period of 6 months before sample collection was used during the selection of subjects for the study. No major illness was reported during the sampling, and those who took antibiotics during the sampling period were excluded from the study. |
| 3.6 | Analytic sample size | Explain how the final analytic sample size was calculated, including the number of cases and controls if relevant, and reasons for dropout at each stage of the study. This should include the number of individuals in whom microbiome sequencing was attempted and the number in whom microbiome sequencing was successful. | STORMS | Consider use of a flow diagram (see template at https://stormsmicrobiome.org/figures). Also state sample size in abstract.  If power analysis was used to calculate sample size, describe those calculations. | Yes | Method, Page 23-24  Results, Page 6 -17  Figure legends  Supplementary Results, and Suppl. Figure legends. |
| 3.7 | Longitudinal Studies | For longitudinal studies, state how many follow-ups were conducted, describe sample size at follow-up by group or condition, and discuss any loss to follow-up. | STORMS | If there is loss to follow-up, discuss the likelihood that drop-out is associated with exposures, treatments, or outcomes of interest. | Yes | Temporal sampling over three different seasons, hot-humid summer (S1), autumn (S2) and cold-dry winter (S3), were collected from the study population. A two-day diet recall was recorded during each sampling.  Methods, Page 24  Results, page 6  Supplementary Results, Page 1. |
| 3.8 | Matching | For matched studies, give matching criteria. | Modified STROBE | "Matched" refers to matching between comparable study participants as cases and controls or exposed / unexposed.  Indicate whether participants were individual or frequency matched and in what ratio were they matched (e.g. 1 case to 1 control). | Yes | The Indian study groups were balanced in age, sex, body mass index (BMI), nature of birth, dietary and lifestyle habits.  The three seasonal samples were matched.  Significance in Diet and seasonal influence were calculated by matching the samples of the subjects.  For comparison, a set of European individuals (n=76; data from the HITChip database) matched for age, BMI and gender was used for the study.  .  Result, Pages 10-17  Method, Pages 23-33. |
| 3.9 | Ethics | State the name of the institutional review board that approved the study and protocols, protocol number and date of approval, and procedures for obtaining informed consent from participants. | STORMS |  | Yes | Ethical approval, Page 43  The Institutional Ethical Committee (IEC) of the Institute of Bioresources and Sustainable Development, Imphal, India (approval number IBSD/IEC/2018/003) and the Department of Biotechnology, Government of India approved the study protocol. Furthermore, we obtained informed consent from all the participants and followed the guidelines of the Indian Council of Medical Research (ICMR) (www.icmr.nic.in/ethics_SOP.pdf). |
| 4.0 | Laboratory methods | State the laboratory/center where laboratory work was done. | STORMS | Provide a reference to complete lab protocols if previously published elsewhere such as on protocols.io. Note any modifications of lab protocols and the reason for protocol modifications. | Yes | Lab work was done at Wageningen University and IBSD, mentioned in the Acknowledgement and Author contribution section, Page 42. |
| 4.1 | Specimen collection | State the body site(s) sampled from and how specimens were collected. | MIxS: sample collection device or method; host body site | Use terms from the Uber-anatomy Ontology (https://www.ebi.ac.uk/ols/ontologies/uberon) to describe body sites in a standardized format. | Yes | Fecal sample (self-collected) with informed consent.  Methods, pages 24-25.  Results, page 6 |
| 4.2 | Shipping | Describe how samples were stored and shipped to the laboratory. | STORMS | Include length of time from collection to receipt by the lab and if temperature control was used during shipping. | Yes | The self-collected faecal samples were frozen immediately and transported to the laboratory within two hours of collection.    Method, Page 25 |
| 4.3 | Storage | Describe how the laboratory stored samples, including time between collection and storage and any preservation buffers or refrigeration used. | STORMS | State where each procedure or lot of samples was done if not all in the same place.  Include reagent/lot/catalogue #s for storage buffers. | Yes | The faecal samples were kept at -80°C for analysis.  Method, Page 26 |
| 4.4 | DNA extraction | Provide DNA extraction method, including kit and version if relevant. | MIxS: nucleic acid extraction | If any DNA quantification methods were used prior to DNA amplification or at the pooling step of library preparation, state so here. | Yes | Repeated bead beating method (Salonen et al, 2010).  Method, Page 26 |
| 4.5 | Human DNA sequence depletion or microbial DNA enrichment | Describe whether human DNA sequence depletion or enrichment of microbial or viral DNA was performed. | STORMS |  | No |  |
| 4.6 | Primer selection | Provide primer selection and DNA amplification methods as well as variable region sequenced (if applicable). | MIxS: pcr primers |  | Yes | Method, page 23-28,  Supplementary information, Table S8 |
| 4.7 | Positive Controls | Describe any positive controls (mock communities) if used. | STORMS | If used, should be deposited under guidance provided in the 8.X items. | Yes | The DNA extracted from the microbial strains of *Escherichia coli* DH5α, *Bacteroides thetaiotaomicron* DSM 2079, *Ruminococcus gnavus* ATCC 29149,  *Methanobrevibacter smithii* DSM 274, *Ligilactobacillus plantarum* WCFS1, *Bacillus subtilis* ATCC6051,  *Prevotella copri* DSM-18205, *Bifidobacterium longum* subsp. longum DSM 20219, *Bacteroides fragilis* were used as a positive control for qPCR assay, Supplementary information,  Table S8. |
| 4.8 | Negative Controls | Describe any negative controls if used. | STORMS | If used, should be deposited under guidance provided in the 8.X items. | Yes | Negative controls were used in the qPCR and PCR for HITChip. |
| 4.9 | Contaminant mitigation and identification | Provide any laboratory or computational methods used to control for or identify microbiome contamination from the environment, reagents, or laboratory. | STORMS | Includes filtering of reagents and other steps to minimize contamination. It is relevant to state whether the specimens of interest have low microbial load, which makes contamination especially relevant. | Yes | Standard sterile collection method followed. The reagents used for the study were tested for microbial contamination by DNA extraction and bacterial-specific qPCR assay. |
| 4.10 | Replication | Describe any biological or technical replicates included in the sequencing, including which steps were replicated between them. | STORMS | Replication may be biological (redundant biological specimens) or technical (aliquots taken at different stages of analysis) and used in extraction, sequencing, preprocessing, and/or data analysis. | Yes | Method, page 23-32  HITChip, a phylogenic microarray analysis of three seasonal faecal samples from Indian subjects (214 samples), was performed with duplicates. HPLC and qPCR analysis was performed in triplicate. All replicates were successful. |
| 4.11 | Sequencing strategy | Major divisions of strategy, such as shotgun or amplicon sequencing. | MIxS: sequencing method | For amplicon sequencing (for example, 16S variable region), state the region selected. State the model of sequencer used. | Yes | Method, Pages 25-26,  & Pages 28-29.  Microarray (HITChip), probes targeting 16S rRNA gene and taxa-specific qPCR assay were used for this study.  Supplementary Information,  1.2 Methodology for assessing the bacterial community structure of fermented foods (*Hawaijar* and *Dahi*) by Illumina-MiSeq amplicon sequencing,  Page 3. |
| 4.12 | Sequencing methods | State whether experimental quantification was used (QMP/cell count based, spike-in based) or whether relative abundance methods were applied. | STORMS | These include read length, sequencing depth per sample (average and minimum), whether reads are paired, and other parameters. | Yes | Supplementary Information,  Table S4 and Table S5, Bacterial community structure of *Dahi* (fermented milk) and *Hawaijar* analysed by Illumina-MiSeq amplicon sequencing  Method, Page 25  Microarray (HITChip), probes targeting 16S rRNA gene and taxa-specific qPCR assay were used for this study. |
| 4.13 | Batch effects | Detail any blocking or randomization used in study design to avoid confounding of batches with exposures or outcomes. Discuss any likely sources of batch effects, if known. | STORMS | Sources of batch effects include sample collection, storage, library preparation, and sequencing and are commonly unavoidable in all but the smallest of studies. | Yes | The faecal sample DNA/ metabolite extraction was kept blind without knowing the sample group in the study. |
| 4.14 | Metatranscriptomics | Detail whether any mRNA enrichment was performed and whether/how retrotranscription was performed prior to sequencing. Provide size range of isolated transcripts. Describe whether the sequencing library was stranded or not. Provide details on sequencing methods and platforms. | STORMS | Provide details on any internal standards which may have been used as well as parameters and versions of any software or databases used. | No |  |
| 4.15 | Metaproteomics | Detail which protease was used for digestion. Provide details on proteomic methods and platforms (e.g. LC-MS/MS, instrument type, column type, mass range, resolution, scan speed, maximum injection time, isolation window, normalised collision energy, and resolution). | STORMS | Provide details on any internal standards which may have been used as well as parameters and versions of any software or databases used. | No |  |
| 4.16 | Metabolomics | Specify the analytic method used (such as nuclear magnetic resonance spectroscopy or mass spectrometry). For mass spectrometry, detail which fractions were obtained (polar and/or non-polar) and how these were analyzed. Provide details on metabolomics methods and platforms (e.g. derivatization, instrument type, injection type, column type and instrument settings). | STORMS | Provide details on any internal standards which may have been used as well as parameters and versions of any software or databases used. | Yes | Method, Page 26  LC-HRMS was used for the metabolite profiling of faecal samples  Taurocholate was used as an internal standard. |
| 5.0 | Data sources/  measurement | For each non-microbiome variable, including the health condition, intervention, or other variable of interest, state how it was defined, how it was measured or collected, and any transformations applied to the variable prior to analysis. | MIxS: host disease status | State any sources of potential bias in measurements, for example multiple interviewers or measurement instruments, and whether these potential biases were assessed or accounted for in study design.  Use terms from a standardized ontology such as the Experimental Factor Ontology (https://www.ebi.ac.uk/efo/) to describe variables of interest in a standardized format. | Yes | A questionnaire was used to collect health and diet recall information. |
| 6.0 | Research design for causal inference | Discuss any potential for confounding by variables that may influence both the outcome and exposure of interest. State any variables controlled for and the rationale for controlling for them. | STORMS | For causal inference, this item refers to describing the assumptions that would be required to draw causal inferences from observational data. See Vujkovic-Cvijin, I., Sklar, J., Jiang, L. et al. Host variables confound gut microbiota studies of human disease. Nature 587, 448–454 (2020). https://doi.org/10.1038/s41586-020-2881-9 for more details on confounding in observational microbiome studies.  For example, hypothesized confounders may be controlled for by multivariable adjustment. Consider using a directed acyclic graph (DAG) to describe your causal model and justify any variables controlled for. DAGs can be made using [www.dagitty.net](http://www.dagitty.net/). | Yes | As suggested by Reviewer #1, we have done confounder analysis by Linear Regression (IBM SPSS Statistics, Version 29.0.2.0) to find out the significant effects of other factors apart from the consumption of the two fermented foods, included the results section, Results pages 7- 23.  Method section, page 33 |
| 6.1 | Selection bias | Discuss potential for selection or survival bias. | STORMS | Selection bias can occur when some members of the target study population are more likely to be included in the study/final analytic sample than others. Some examples include survival bias (where part of the target study population is more likely to die before they can be studied), convenience sampling (where members of the target study population are not selected at random), and loss to follow-up (when probability of dropping out is related to one of the things being studied). | Yes | Method, Page 23-24  The subjects of four diet groups were randomly selected from the list of qualified subjects with the selection criteria. |
| 7.0 | Bioinformatic and Statistical Methods | Describe any transformations to quantitative variables used in analyses (e.g. use of percentages instead of counts, normalization, rarefaction, categorization). | STORMS | If a variable is analyzed using different transformations, state rationale for the transformation and for each analyses which version of the variable is used.  In case of any complex or multistep transformations, give enumerated instructions for reproducing those transformations. | Yes | Method, Pages 23-34  Log transformation, fold change, relative abundance, absolute abundance, ratios, etc. all mentioned in the Methods and Results, Figures and Tables. |
| 7.1 | Quality Control | Describe any methods to identify or filter low quality reads or samples. | MIxS: sequence quality check | If samples were excluded based on quality or read depth, list the criteria used, the number of samples excluded, and the final sample size after quality control. | Yes | Method, Page 23-34  In Microarray (HITChip), probes targeting 16S rRNA gene by cy3 and cy5 signal correlation are used as quality control.  For taxa-specific qPCR assays, the standard graph slope R2-value is used for quality control.  For Illumina MiSeq amplicon sequencing the read numbers are considered for the quality. |
| 7.2 | Sequence analysis | Describe any taxonomic, functional profiling, or other sequence analysis performed. | MIxS: feature prediction; similarity search method |  | Yes | Method, Page 25  The HITChip probe signals were assigned to three phylogenetic levels. Level 1: order-like 16S rRNA gene sequence groups and level 2: genus-like with sequence similarity of >90%.  SILVA database was used for the generation of OTU tables at different taxonomic levels using QIIME2, for the 16S rRNA amplicon sequencing, in Supplementary methods. |
| 7.3 | Statistical methods | Describe all statistical methods. | Modified STROBE | Describe any statistical tests used, exploratory data analysis performed, dimension reduction methods/unsupervised analysis, alpha/beta metrics, and/or methods for adjusting for measurement bias.  If multiple statistical methods are possible, discuss why the methods used were selected.  If a multiple hypothesis testing correction method was used, describe the type of correction used.  State which taxonomic levels are analyzed. | Yes to all | Methods, Pages 23-32  Results, Pages 6-17  Statistics & Reproducibility section in the Methods |
| 7.4 | Longitudinal analysis | If the study is longitudinal, include a section that explicitly states what analysis methods were used (if any) to account for grouping of measurements by individual or patterns over time. | STORMS |  | Yes | Method, Page 23  Results, Pages 6  Discussion, Page 17  Three-time interval faecal samples (hot-humid summer, autumn and dry-winter) were analysed. |
| 7.5 | Subgroup analysis | Describe any methods used to examine subgroups and interactions. | STROBE |  | Yes | Method, page 23-31  Diet and seasonal effects, Co-occurrence network analysis for interaction. |
| 7.6 | Missing data | Explain how missing data were addressed. | STROBE | "Missing data" refers to participant measurements such as covariates, exposures, outcomes, or time points that should have been collected but were not, not to zeros in taxonomic abundance tables or data points not applicable to that observation. | Yes | Method, pages 32-33.  Supplementary Results, Page 2  Among the 85 subjects targeted, seven subjects were declined during the first sampling, and seven subjects were discontinued after the first sampling due to the health condition, medication/antibiotic intake or deviation from the eligibility criteria. Seven seasonal samples were missed due to the subject's travel during the collection time.  Missing data in diet recall data were mentioned in the Metadata file provided in the Source Data. |
| 7.7 | Sensitivity analyses | Describe any sensitivity analyses. | STROBE |  | Yes | Method, Pages 32-33.  Two dyes were used for microarray sensitivity analysis for all samples analysed by HITChip.  Frequent injections of known standards with known concentrations for HPLC and LC-HRMS were used to check the sensitivity. |
| 7.8 | Findings | State criteria used to select findings for reporting. | STORMS | For example, false discovery rate with total number of tests, effect size threshold, significance threshold, microbes of interest. | Yes | Throughout the manuscript, figures and Tables. |
| 7.9 | Software | Cite all software (including read mapping software) and databases (including any used for taxonomic reference or annotating amplicons, if applicable) used. Include version numbers. | Modified STREGA | Installed packages, add-ons or libraries should be stated and cited in addition to the software used.  All parameters employed that differ from the default of that software/version should be provided.  This is in addition to, not a replacement for, publishing of code as outlined in the section Reproducible Research. | Yes | Method, Pages 23-26  Agilent microarray scanner feature extraction software, version 9.5 ([http://www.agilent.com](http://www.agilent.com/)) MySQL database management system (<http://www.mysql.com/>); Multivariate analysis using PAST v3.2266 and Canoco software v4.52 (Wageningen University, The Netherlands);  Boxplot using BoxPlotR (http://shiny.chemgrid.org/boxplotr/) and violin plots using PAST 3.22;  Co-occurrence network analysis 'Gephi' (Version 0.8.2-beta) (https://gephi.org/‎);  R package (v3.1.3) (gplot, svDialogs, vegan, random forest, beanplot, i-graph, microbiome, mia); IBM SPSS Statistics, Version 29.0.2.0;  The MS and MSMS spectra analysis (SIEVE 2.2, Thermo Fisher Scientific);  High-performance liquid chromatography (Accela, Thermo).  qPCR assays in Biorad CFX384 (Biorad, USA) and ABI 7500 instrument (Life Technologies, USA).  Microsoft Excel 2010. |
| 8.0 | Reproducible research | Make a statement about whether and how others can reproduce the reported analysis. | STORMS | Any protected information that has been excluded or provided under controlled access should be listed along with any relevant data access procedures. "On request from authors" is not sufficiently detailed; formal data access procedures and conditions should be defined.  If data are unavailable, state so clearly.  Consider using a specialized rubric for reproducible research (such as:<https://mbio.asm.org/content/9/3/e00525-18.short)>.  Consider preregistering the study protocol (such as o[n osf.](http://osf.io/)io or<https://plos.org/open-science/preregistration/).> | Yes | Yes, both sample metadata and The HITChip microarray data are available in HITChip Atlas under the project name “CREST study” maintained by the Laboratory of Microbiology, Wageningen University & Research. The HITChip data are available via Zenodo (https://doi.org/10.5281/zenodo.14369940) under an MIT license.  Source Data: Provided as a separate Excel file, describing the Source Data of figures.  Yes, the Reproducibility statement is in the Methods section.  No, we did not preregister the study. |
| 8.1 | Raw data access | State where raw data may be accessed including demultiplexing information. | STORMS | Robust, long-term databases such as those hosted by NCBI and EBI are preferred. If using a private repository, provide rationale. | Yes | Data availability statement, Page 34  Data sets used in this study, including the probe-level Human Intestinal Tract phylogenetic microarray (HITChip) data, the associated sample metadata and the derived higher-level genus and phylum level taxonomic abundance tables, qPCR data on absolute abundance and chemical profiling data are available with a permanent DOI via Zenodo (<https://doi.org/10.5281/zenodo.14424024>).  The microbiota profiling data for the fermented foods (16S rRNA gene amplicon sequencing data) is available in NCBI-SRA data (accession number: PRJNA1191989). |
| 8.2 | Processed data access | State where processed data may be accessed. | STORMS | Unfiltered data should be provided.  Robust, long-term databases such as those hosted by NCBI and EBI-EMBL are preferred. Repositories like zenodo (https://zenodo.org/) or publisso (https://www.publisso.de/en/working-for-you/doi-service/)  can be used to provide a DOI and long-term storage for processed datasets, even those which cannot be published openly. | Yes | The processed data are available in the Supplementary Information, Source Data, and Supplementary Data files. |
| 8.3 | Participant data access | State where individual participant data such as demographics and other covariates may be accessed, and how they can be matched to the microbiome data. | STORMS | If re-categorized, transformed, or otherwise derived variables were used in the analysis, these variables or code for deriving them should be provided.  Examples of how participant data can be matched to microbiome data are: using the same set of anonymized identifiers, or using different anonymized identifiers but providing a map.  Provided data should be sufficient to independently replicate the current analysis. | Yes | The variability of individual recruited subjects (age, sex, BMI), habitual diet and lifestyles, and 48 h diet recall data retrieved from the questionnaire is provided separately as a Metadata file in the Source Data and Supplementary Data).  The participants were anonymised by providing aggregated data with no indirect identifier information. |
| 8.4 | Source code access | State where code may be accessed. | STORMS | If a standard or formalized workflow was employed, reference it here. | Yes | Custom R scripts are available at available via Zenodo (https://doi.org/10.5281/zenodo.14369940) under an MIT license. |
| 8.5 | Full results | Provide full results of all analyses, in computer-readable format, in supplementary materials. | STORMS | For example, any fold-changes, p-values, or FDR values calculated, provided as a spreadsheet.  Use a machine-readable, plain-text format such as csv or tsv. | Yes | Supplementary information, with results, figures and tables are provided. Source Data for full results with readable R scripts are provided via Zenodo (https://doi.org/10.5281/zenodo.14369940) |
| **Results** | | | | | | |
| 9.0 | Descriptive data | Give characteristics of study participants (e.g. dietary, demographic, clinical, social) and information on exposures and potential confounders. | STROBE | Typically reported in a table included in the paper or as a supplementary table. Indicate number of participants with missing data for each variable of interest.  This includes environmental and lifestyle factors that may affect the relationship between the microbiome and the condition of interest. Participant diet and medication use should be summarized, if known.  At minimum, age and sex of all participants should be summarized. | Yes | Supplementary information, Table S1, about the Metadata of the study subjects in each categorized group and supplementary results about the Summary of the results obtained from the questionnaire administered to the Indian study population about their dietary and lifestyle habits during the seasonal sampling.  Supplementary Data,  The variability of individual recruited subjects (age, sex, BMI) is provided in the Source Data, and the habitual diet and lifestyle data retrieved from the questionnaire are provided separately as Supplementary Data. |
| 10.0 | Microbiome data | Report descriptive findings for microbiome analyses with all applicable outcomes and covariates. | STORMS | This includes measures of diversity as well as relative abundances. These descriptive findings should be reported both for the sample overall and for individual groups. | Yes | Results, pages 6-17 |
| 10.1 | Taxonomy | Identify taxonomy using standardized taxon classifications that are sufficient to uniquely identify taxa. | STORMS | If not using full taxonomic hierarchy, make sure it is clear whether names stated are species, genera, family, etc.  Italicize genus/species pairs. Consult journal guidelines or standardized references on taxonomic nomenclature. For instance,<https://wwwnc.cdc.gov/eid/page/scientific-nomenclature> | Yes | Results, pages 6-17 |
| 10.2 | Differential abundance | Report results of differential abundance analysis by the variable of interest and (if applicable) by time, clearly indicating the direction of change and total number of taxa tested. | STORMS | If there are more than two groups, include omnibus (multigroup) test results if applicable to the research question.  If applicable, reported effect sizes should include a measure of uncertainty such as the confidence interval. | Yes | Results, pages 6-17 |
| 10.3 | Other data types | Report other data analyzed--e.g. metabolic function, functional potential, MAG assembly, and RNAseq. | STORMS |  | Yes | Metabolite profile data  Results, page 9 |
| 10.4 | Other statistical analysis | Report any statistical data analysis not covered above. | STORMS | This could include subgroup analysis, sensitivity analyses, and cluster analysis.  Visualizations should be easily interpretable and colorblind-friendly. The caption and/or main text should provide a detailed description of visualizations for visually-impaired readers. | Yes | Bimodality analysis, Principal response curve (PRC) analysis,  Results, Page 16 |
| **Discussion** | | | | | | |
| 11.0 | Key results | Summarise key results with reference to study objectives | STROBE |  | Yes | Discussion, pages 17-23 |
| 12.0 | Interpretation | Give a cautious overall interpretation of results considering objectives, limitations, multiplicity of analyses, results from similar studies, and other relevant evidence. | STROBE | Define or clarify any subjective terms such as "dominant," "dysbiosis," and similar words used in interpretation of results.  When interpreting the findings, consider how the interpretation of the findings may be summarized or quoted for the general public such as in press releases or news articles.  If causal language is used in the interpretation (such as "alters," "affects," "results in," "causes," or "impacts"), assumptions made for causal inference should be explicitly stated as part of 6.0 and 13.0.  Distinguish between function potential (ie inferred from metagenomics) and observed activity (ie metatranscriptomic, metabolomic, proteomic) if discussing microbial function. | Yes | Results,  Figures 1-8.  Discussion section  For a clear interpretation of significant results, we included statistics for each statement in the text. We used simplified figures for easy interpretation of the significant results. |
| 13.0 | Limitations | Discuss limitations of the study, taking into account sources of potential bias or imprecision. | STROBE | Also consider limitations resulting from the methods (especially novel methods), the study design, and the sample size. | Yes | Discussion, page 23  We acknowledge that the small sample size is a limitation of our work in the Discussion section concluding remarks, in lines 579-583, page 23.  Only bacteria and Archaea were taken into account for the study. As it is a self-reported consumption pattern of fermented foods over a long period of 10 years, the changes in the frequency and quantity of consumption over the season may make a potential bias or imprecision. |
| 13.1 | Bias | Discuss any potential for bias to influence study findings. | STORMS | May include sampling method, representativeness of study participants, or potential confounding. | Yes | Discussion, Page 17  Though we maintained a diet recall for the last two days before sampling, the seasonal dietary differences between the subjects may cause a bias. We corrected the confounder while reporting the diet effects. |
| 13.2 | Generalizability | Discuss the generalisability (external validity) of the study results | STROBE | To what populations or other settings do you expect the conclusions to generalize? | Yes | The bimodal distribution (Cluster-P with *Prevotella* enrichment; Cluster-B/R with *Bifidobacterium/Ruminococcus* enrichment) visualized in Indian and European study population during present study, was also visualised when the gut microbiota across geography was put together, as a difference between industrialised countries compared with traditional societies.  Discussion, page 22 |
| 14.0 | Ongoing/future work | Describe potential future research or ongoing research based on the study's findings. | STORMS |  | Yes | Discussion, pages 21-22  Seasonal impact of fermented foods on gut microbiota, particularly, fermented soybean on *Bifidobacterium* during winter and Fermented milk on *Catenibacterium* during summer.  The difference in the bimodal distribution (Cluster-P with *Prevotella* enrichment; Cluster-B/R with *Bifidobacterium/Ruminococcus* enrichment) in industrialised vs traditional population or developed vs underdeveloped population, functional consequences of this ecological difference in the gut microbiota, is a potential area for future study. |
| **Other information** | | | | | | |
| 15.0 | Funding | Give the source of funding and the role of the funders for the present study and, if applicable, for the original study on which the present article is based | STROBE |  | Yes | Acknowledgements, Page 43  This research was supported by the Department of Biotechnology (DBT), Ministry of Science and Technology, Government of India through the DBT-CREST award (Department of Biotechnology Cutting-edge Research Enhancement and Scientific Training Award) to Dr. K.Jeyaram (BT/IN/CREST-Awards/44/KJ/2010-11). The financial support of the Institute of Bioresources and Sustainable Development (IBSD), India also gratefully acknowledged. The Spinoza grant to Prof. Willem M. de Vos is gratefully acknowledged for the HITChip analyses. Dr. Leo Lahti was additionally supported by the Academy of Finland (decision 295741, 330887). |
| 15.1 | Acknowledgements | Include acknowledgements of those who contributed to the research but did not meet critera for authorship. | STORMS | For general guidelines on authorship, see [http://www.icmje.org](http://www.icmje.org/) and<https://www.elsevier.com/authors/journal-authors/policies-and-ethics/credit-author-statement> | Yes | Acknowledgements, Page 43 |
| 15.2 | Conflicts of Interest | Include a conflicts of interest statement. | STORMS |  | Yes | Competing interest statement, page 44 |
| 16.0 | Supplements | Indicate where supplements may be accessed and what materials they contain. | STORMS |  | Yes | Supplementary Information (42 pages) referred to throughout the text: It includes Supplementary Results, Supplementary Figures (Fig. S1–S17), Supplementary Tables (Tables S1-S8) and Supplementary References. |
| 17.0 | Supplementary data | Provide supplementary data files of results with for all taxa and all outcome variables analyzed. Indicate the taxonomic level of all taxa. | STORMS | Depending on the analysis performed, examples of the supplemental results included could be mean relative abundance, differential abundance, raw p-value, multiple hypothesis testing-adjusted p-values, and standard error.  All discussed taxa should include the taxonomic level (e.g. class, order, genus). | Yes | Supplementary Data, the variability in habitual diet and lifestyle data retrieved from the questionnaire are provided separately as an Excel file. |
